# Proteomic profiling of small extracellular vesicles from bovine nucleus pulposus cells

**DOI:** 10.1371/journal.pone.0324179

**Published:** 2025-05-29

**Authors:** Ankita Samanta, Mi-Jeong Yoo, Jin Koh, Sina Charlotte Lufkin, Thomas Lufkin, Petra Kraus

**Affiliations:** 1 Department of Biology, Clarkson University, Potsdam, New York, United States of America; 2 The Interdisciplinary Center for Biotechnology Research, University of Florida, Gainesville, Florida, United States of America; Hong Kong Metropolitan University, HONG KONG

## Abstract

Small extracellular vesicles (small EV) are a conserved means of communication across the domains of life and lately gained more interest in mammalian non-cancerous work as non-cellular, biological therapeutic with encouraging results in recent studies of chronic degenerative diseases. The nucleus pulposus (NP) is the avascular and aneural center of an intervertebral disc (IVD), home to unique niche conditions and affected in IVD degeneration. We investigated autologous and mesenchymal stem cell (MSC) small EVs for their potential to contribute to cell and tissue homeostasis in the NP niche via mass spectrometric proteome and functional enrichment analysis using adult and fetal donors. We compared these findings to published small EV databases and MSC small EV data. We propose several mechanisms associated with NP small EVs: Membrane receptor trafficking to modify signal responses promoting niche homeostasis; Redox and energy homeostasis via metabolic enzymes delivery; Cell homeostasis via proteasome delivery and immunomodulation beyond an association with a serum protein corona. The proteome signature of small EVs generated by NP parent cells is similar to previously published small EV data, yet with a focus on supplementing anaerobic metabolism and redox balance while contributing to the maintenance of an aneural and avascular microniche.

## Introduction

Lower back and neck pain present global health issues as they are significant causes of disability worldwide. It is the most frequent cause of worker’s compensation, lost working time, and decreased productivity and as such associated with a large socioeconomic burden [1,2]. Even if the etiology of most cases of back pain remains unknown, intervertebral disc degeneration (IVDD) is one of the most common factors [3,4]. IVDD principally impacts the intervertebral disc (IVD), resulting in structural modifications and functional impairments within the spinal column [5–7]. Addressing this complex and multidimensional illness with conventional approaches hampers clinical success. Therefore identifying factors that promote cell and tissue homeostasis prior to disc degeneration remains important [6,8].

**Fig 1:**
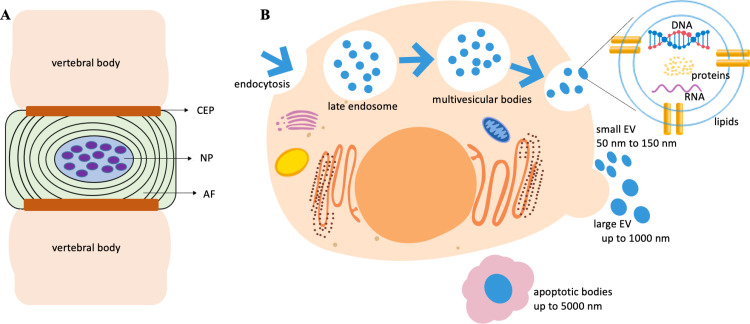
The intervertebral disc (IVD) and the generation of small extracellular vesicles. A) Schematic representation of an IVD with a central nucleus pulposus (NP) surrounded by an annulus fibrosus (AF) present in between the cartilaginous endplates. B) Small extracellular vesicle (EV) biogenesis and composition. This illustration was created on the Wondershare Edraw Max Platform. (https://edrawmax.wondershare.com/).

The IVD is made up of a central nucleus pulposus (NP) surrounded by an inner and outer annulus fibrosus (AF), which is present in between the cartilaginous endplates (CEPs) (Fig 1A) [9–17]. The AF is mostly made up of collagen type I. Cells of the outer AF appear elongated, thin, and parallel to the collagen lamellae, while the inner AF looks fibrocartilaginous [10,13,14,18–21]. The NP is of great functional importance and by volume the largest tissue type in the IVD. The NP has a relatively loose collagen network, is highly hydrated, and exhibits hydrostatic behavior under load when non-degenerate [6,10,14,18]. Proteoglycans (PG), in particular aggrecan (Acan) with its high anionic glycosaminoglycan (GAG) content, specifically chondroitin sulfate and keratan sulfate, comprise 5–15% of the weight of wet tissue. Their charges ensure the swelling pressure to maintain disc height and turgor under compressive load by attracting water molecules into the disc [10,19,22,23]. One of the earliest changes in IVDD is PG-loss and consequentially reduced water retention, resulting in decreased disc height and flexibility [10]. NP cells originate from the notochord (NC), a transient embryonic structure [13,24,25]. In the mature NP, unlike most tissues, few cells are embedded in a vast amount of extracellular matrix (ECM) [14,18]. In some species like rodents, and pigs NC cells are retained into mature adulthood where they continue to generate PGs and collagen [13,26,27]. In human and bovine IVDs, these NC cells are replaced by chondrocyte-like cells around the age of ten years [20,25,28–37]. Similar to the bovine model, the adult human NP has a low cell density, with cells occupying less than 0.5% of the tissue volume [38]. With aging, the density of active NP cells declines further, presumably due to niche conditions [39–41]. In general, bovine coccygeal IVDs are considered similar to healthy human IVDs [42–45]. This makes the bovine IVD a suitable model to study cell homeostasis in the adult. Only recently, more information has become available on the proteome and transcriptome of healthy or degenerated IVDs from various species [46–55] and more potential biomarkers defining NP and AF cells joined those previously established on the transcriptome level [47,52,56,57]. Contributions to this data would refine a characterization of the heterogeneous NP cell population and further define hallmarks to maintain or restore IVD tissue.

Regenerative medicine aims to repair ailing tissues and organs. Related mesenchymal stem cell (MSC) therapy could restore disc height and NP-MSCs have been identified in both degenerative and normal IVD tissues, showing a remarkable resilience to acidic conditions *in vitro* compared to other MSCs, alongside increased expression levels of key ECM components such as PGs and collagen II [55,58]. Transplanting NP-MSCs into the IVD for differentiation into NP cells is one regenerative approach for IVDD [59]. However, limitations with cell delivery and the harsh, hypoxic microenvironment in the mature NP challenges their viability *in vivo*, making successful long term therapy uncertain [10,38,60–64]. Another strategy pursued in tissue regeneration is the activation of endogenous progenitor cells. It has been acknowledged that the MSC secretome plays an important part in immune modulation and regeneration [65,66]. The homeostasis of a microenvironment is naturally maintained via effective cell-cell communication [67]. The healthy NP is avascular and aneural [17,38,68,69] and possibly depended on small extracellular vesicle (EV) exchange for communication between isolated NP cells in the ECM. Small EVs with unique proteins, lipids, and nucleic acids specific to their parent cells are now recognized as intercellular communication methods [70]. Small EVs may interact with target cells, conveying cargo of bioactive molecules from parent to recipient cells thereby influencing their behavior and phenotypic traits by impacting cellular processes such as cell proliferation, differentiation, immune responses, and gene expression [71–73]. Small EVs play significant roles in disease progression, including cancer and neurodegenerative disorders [74]. The development of large-scale “-omics” technologies has improved our understanding of the secretome, including EVs with specific cargo of proteins and nucleic acids [67,75]. Although the classification of EVs constantly changes [76] they usually fall into one of three categories based on size (Fig 1B): Apoptotic bodies (up to 5000nm), ectosomes (up to 1000nm), and small EVs (50–150nm), previously referred to as exosomes [72,77]. Recent guidelines recommend a nomenclature limiting the term “exosome” to only those small EVs demonstrated as generated via multivesicular bodies (MVB) [76]. Most cell types including resident stem- and progenitor cells produce small EVs, and their release into body fluids and culture media has sparked interest in the identification of cell or disease-specific biomarkers, similar to research in oncology [78–83]. IVD small EV biomarkers could refine IVD cell phenotyping. Size, composition, functional influence on recipient cells, and the biological origin of small EVs all contribute to their heterogeneity [67,72]. The acellular nature of small EVs offers novel opportunities for IVDD therapy beyond constrains of stem cell therapies such as tumorigenesis, cell rejection or cell survival [75,84,85]. As part of the cell secretome, small EVs maintain the therapeutic benefits of their parent cell and protect their cargo via a phospholipid membrane [75,86]. Previous small EV research focused on the function and composition of NC cell derived EV microRNAs (miRNAs). These small non-coding RNAs are studied extensively for their therapeutic potential in other domains [87]. NC cell-derived EVs enhanced DNA and GAG content in human NP cell micro-aggregates compared to untreated controls, although the underlying mechanism and associated EV content were not determined [88]. Limited research has been conducted on IVD-derived EVs in the context of IVD homeostasis, largely focusing on benefits of MSC derived small EVs [85,86,89–96]. In a recent study, EVs derived from NP cells promoted the proliferation of degenerated NP cells and reduced senescence *in vitro*, while attenuating IVDD *in vivo* [97], in another very recent study the cargo of small EVs generated by parent cells derived from degenerated human IVDs was investigated [98]. Identifying the best suited parent cell could be essential. Based on IVD location and anatomy, harvesting autologous cells of a healthy NP or AF could inflict organ damage (Fig 1). Adipose tissue is most readily accessible and has been a source for autologous MSC treatments for other conditions [99–101]. However, dysfunctional fat metabolism and chronic activation of ERK/MAPK signaling has been linked to tissue aging in a number of animal models including humans [102–104]. Investigating small EV cargo from NP and other parent cells could provide new insight into cell-cell communication and signaling cascades that maintain IVD homeostasis and point to novel strategies to prevent, delay or alleviate IVDD [6,75,89]. To address the potential of parent cell sources for small EV production we investigated the proteome of small EVs generated by autologous IVD and adipose tissue derived cells from healthy adult and fetal bovine donors and compared our novel data sets to existing MSC small EV data. Through gene ontology (GO) annotation and functional enrichment analyses we identified similarities and differences in the protein profiles of these small EVs. We further conducted quantitative small EV proteomics to compare NP small EVs with their parent cells. We found consistency with existing small EV data alongside proteins and molecular pathways relevant for NP cell homeostasis to serve as targets for the development of future IVDD interventions.

## Materials and methods

### Ethics statement

No human materials or subjects were used, no IRB required. No live animals were used. The work has been reported in line with the ARRIVE guidelines 2.0 and is covered under IACUC protocol/approval number 19−04, approved 10/24/2021. Project title: Analysis of Gene Expression. Institution: Clarkson University.

### Isolation and culture of primary bovine adipose and IVD cell lines

**Fig 2:**
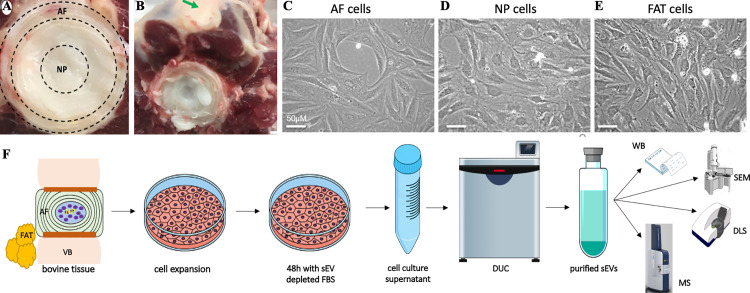
Isolation of parent cell lines and small EVs from bovine tissue. A) Areas of a bovine coccygeal IVD for NP and AF tissue harvest are indicated by dotted lines. B) The green arrow points to subcutaneous adipose tissue after the skin was removed. C) AF cells in culture. D) NP cells in culture. E) Adipose tissue derived cells in culture. AF: Annulus fibrosus; FAT: Adipose tissue; NP: Nucleus pulposus. F) Simplified illustration of the workflow for small EV harvesting and downstream analysis. AF: Annulus fibrosus; DLS: Dynamic light scattering; DUC: Differential ultracentrifugation; EVs: Extracellular vesicles; FAT: Adipose tissue; FBS: fetal bovine serum; MS: Mass spectrometry; NP: Nucleus pulposus; SEM: Scanning electron microscopy; VB: Vertebral body; WB: Western blot.

Tails from skeletally mature, healthy adult cows were collected fresh as waste product from local abattoirs, stored on ice and immediately transported to the laboratory for dissection (Fig 2A and 2B). Skin, fat and skeletal muscle were removed, prior to isolating coccygeal IVDs flanking the caudal vertebras (cav) from the mid-tail section for tissue culture, typically cav7 to cav11. More proximal IVDs (>cav11) were fixed in 4% paraformaldehyde (PFA) for reference with histological stains like Mallory’s tetrachrome stain or RNA in situ hybridization (RISH) carried out as previously described (S1 Fig) [57,105]. Primary bovine cell-lines were isolated from finely minced tissue pieces of adult and fetal central NPs (referred to as NP), outer AFs (referred to as AF) and adipose tissues (referred to as FAT). The inner AF or transition zone of the IVD was discarded. Primary bovine cell-lines separated by tissue type and donor animal were maintained in Dulbecco’s Modified Eagles Medium (DMEM, GIBCO) with 10% (v/v) heat inactivated fetal bovine serum (HI-FBS performance, #16140071 (USA) GIBCO) and trypsinized with 0.05% Trypsin/EDTA (GIBCO) once 80–90% confluent as previously described [31,88] (Fig 2C–2E). Cell lines isolated in that manner continued to express established cell markers (S1 Fig) [105]. Cells were aliquoted and stored in liquid nitrogen at passage (p) two or three. Culture media components and conditions were kept consistent for all cell lines and would therefore be comparable.

### Small EV isolation from primary IVD cells

For small EV harvest cells were expanded to ~70% confluency in 20 x 150 mm culture dishes, washed three times with 1 × phosphate-buffered saline (PBS), then cultured in a total of 300 ml DMEM with 10% (v/v) small EV-depleted FBS (ED-FBS #A27208-03 (USA,GIBCO). After 48 h at 37˚C under normal oxygen and 5% CO_2_ supernatants were harvested for small EV isolation. A total cell count was estimated from the average of three 150 mm plates. Cells from two plates were lysed in cold 1 × RIPA lysis buffer (50 mM Tris HCl, 150 mM NaCl, 1.0% (v/v) NP40, 0.5% (w/v) sodium deoxycholate, 1.0 mM EDTA, 0.1% (w/v) SDS and 0.01% (w/v) sodium azide, pH 7.4) and stored at −80˚C for further protein analysis. One plate from each cell line was subjected to senescence associated (SA)-β-galactosidase staining for quality control [106]. Cell lines were only included in the study if deemed not senescent. Three biological replicates, NP cell lines TT32, TT33, and TT39 from healthy, adult donors were used for the quantitative proteome analysis of NP small EVs, while NP, AF and FAT cell lines TT39 and fetal (170 days of gestation) NP cells were used for explorative analysis of small EV proteome cargo.

Small EVs for proteome analysis were generally isolated as illustrated (Fig 2F) using differential ultracentrifugation (DUC) [107,108]. Cell debris was eliminated at 300 × *g* in an Eppendorf 5810 centrifuge followed by filtration of the supernatant (0.22 µm cut-off). Larger EVs were removed from the supernatant through DUC at 2000 (2k) × *g* for 30 min at 4ºC in a J2-21 Sorvall ultracentrifuge (rotor JA-20), followed by 10k × *g* for 1 h at 4ºC and 130 k × *g* for 2 h at 4˚C in a Beckman Optima XPN-100 ultracentrifuge (rotor Ti45). Each pellet was resuspended in 1 × PBS and stored at −80˚C. Final pellets were washed with 1 × PBS and repelleted at 130 k × *g* for 2 h at 4˚C before the final resuspension of the small EV fraction in 1 × PBS and storage at −80ºC. Triplicate measurements of protein concentration were conducted with a NanoDrop ND-1000 spectrophotometer (ThermoFisher Scientific) and Bradford assay. All solutions and consumables were nuclease and protease-free. Water or 1 × PBS was filtered with a 0.22 µm cut-off to eliminate dust particles.

### Senescence associated (SA)-β galactosidase staining

Quality of parent cells was assessed via SA-β-Galactosidase staining at the time of small EV harvest and only deemed suitable if less than 10 senescent cells per confluent 150 mm plate were identified by blue staining. Staining was carried out at pH 6 with a staining solution of 1 mg/mL 5-bromo-4-chloro-3-indolyl-beta-D-galacto-pyranoside (X-gal), 5 mM potassium ferricyanide, 5 mM potassium ferrocyanide, 150 mM NaCl and 2 mM MgCl_2_ in 40 mM citric acid/sodium phosphate buffer at pH 6 [106,109].

### Western blot (WB) analysis

Protein samples were separated on a 12% SDS acrylamide gel (Biorad) and transferred to a nitrocellulose membrane (Biorad) at 90 volts for 120 min in 1 × transfer buffer. Membrane blocking was done for 2 h at room temperature, followed by overnight incubation with a primary antibody at 4°C. The membrane was washed 3 × with 1 × Tris buffered saline with Tween 20 (TBST), reblocked for 2 h, and incubated for 2 h at 4°C with the goat anti-rabbit IgG, cross-adsorbed, and horseradish peroxidase (HRP) conjugated secondary antibody (1:5000, ThermoFisher Scientific). Primary antibodies used in this bovine study included CD63 (rabbit, polyclonal (1:1000, Proteintech), TSG101 (rabbit, polyclonal (1:1000, ThermoFisher Scientific)), and Calnexin (rabbit, polyclonal (1:1000, ThermoFisher Scientific). SuperBlock^TM^ PBS Blocking Buffer was used for all blocking and antibody dilutions (ThermoFisher Scientific). The chromogenic 1-Step^TM^ TMB-Blotting Substrate (ThermoFisher Scientific) was used to visualize protein bands.

### Dynamic light scattering (DLS)

Particle analysis was performed by estimating the hydrodynamic diameter of particles present in the different fractions with a Nanobrook 90Plus (Brookhaven Instruments). Samples for DLS were prepared as a suspension. 25 µl of each sample were resuspended well and diluted 1:80 in 1 × PBS before triplicate measurements. After stabilization of laser and temperature of the device, the following parameters were set for reproducibility and standardization: Laser Wavelength: 659 nm, dust cut-off: 40, angle: 90°, temperature: 25°C. Each DLS measurement consisted of 10 acquisitions for 5 sec each. BIC Particle Sizing software from Brookhaven Instruments was used for data acquisition and analysis. Particle size distribution data were presented graphically and numerically, showing the relative particle amount at each size and the cumulative undersize distribution.

### Scanning electron microscopy (SEM)

Small EVs were fixed in 2% PFA, applied to a Formvar coated 400 mesh copper grid (EMS), post fixed with 1% glutaraldehyde, stained for 2 min with UranyLess (EMS) at room temperature, rinsed with deionized water, and imaged on a JEOL JSM 7900F-LV FESEM at 30kV in with a specialty STEM-in-SEM holder. Two technical replicates from one or two biological replicates were generated depending on the cell line.

### Database generation

An extensive library of bovine small EV proteins was generated by integrating data from UniProt [110] and ExoCarta [111]. The ExoCarta proteins were renamed with the UniProt ID mapping tool for subsequent analysis. To identify putative bovine small EV proteins, human small EV protein sequences were searched against bovine protein sequences obtained from UniProt using the Basic Local Alignment Search Tool (BLAST) [112]. The BLAST results were merged with the bovine small EV protein sequences, and all sequences were clustered using CD-HIT v4.6.8 with a sequence identity threshold of 0.95 and a minimum length of 100 [113]. For the explorative bovine small EV proteome profile study, we also used a comprehensive umbilical cord MSC (UCMSC) small EV proteome database [114].

### Small EV protein analysis by liquid chromatography-tandem mass spectrometry (LC-MS/MS)

To deplete high-abundance proteins, extracted small EV proteins were processed with the Pierce albumin and IgG removal kit (P/N 89875 ThermoFisher Scientific) according to the manufacturer’s protocol, which allows the analysis of less abundant proteins. In brief, after the gel slurry was washed, the sample was loaded into the gel slurry and incubated with the orbital rotator for 10 min at room temperature. Then, the flow-through was collected at 10,000 × *g* for 1 min using the spin column. The elution was repeated with the binding and elution process using 75 µl of the binding/wash buffer to obtain the non-redundant proteins.

Depleted small EV proteins were dissolved in protein buffer (8 M Urea, 0.1% SDS, 25 mM Triethylammonium Bicarbonate, pH 8.0) and quantified following a previous method [115]. Protein assays were performed to quantify purified proteins by the EZQ™ Protein Quantification Kit (Thermo Fisher Scientific, San Jose, CA, USA) with the SoftMax Pro Software v5.3 under the SpectraMax M5 (Molecular Devices, LLC). For each sample, a total of 10 μg of protein was reduced with 40 mM DTT, alkylated with 100 mM 2-chloroiodoacetamide, and trypsin-digested (at an enzyme-to-protein ratio (w/w) of 1:100). Tryptic digested peptides were desalted with C18-solid phase extraction (The Nest Group, Inc., Southborough, MA) for the capture of polar and non-polar peptides. Briefly, after equilibrating the cartilage with 1 ml of acetonitrile and 2 ml of water in 0.1% TFA sequentially. The peptide samples were passed over the columns three times before they were washed with 0.1% TFA. The peptides retained on the Sep-Pak C-18 column were eluted in 1 ml of 80% acetonitrile in 0.1% TFA, and the eluant was in the lyophilization.

The hybrid trapped ion mobility-quadrupole time-of-flight mass spectrometer (timsTOF fleX, Bruker Daltonics, Bremen, Germany) with a modified nano-electrospray ion source was interfaced with an ultra-performance EvoSep One LC system (Evosep Biosystem, Odense, Denmark). Briefly, Evotip was wetted with 100 µl of 100% iso-propanol, rinsed with 20 µl of Solvent B (99.9% acetonitrile and 0.1% formic acid (v/v)), equilibrated with 20 ul of 0.1% formic acid (v/v), loaded with 200 ng of digested peptides, and subsequently washed with 20 ul of 0.1% formic acid (v/v) using centrifugal force at 700 x *g* for 1 min. 100 μl of 0.1% formic acid were added to Evotip to prevent drying. Samples were injected into the Bruker timsTOF fleX MS coupled with the Evosep One instrument (Evosep Biosystems). The standard preset method of 15 SPD was used with the EV1106 Endurance column (Evosep One Biosystem) with 200 ng injection. The spectrum library was produced in the data-dependent mode with Parallel Accumulation Serial Fragmentation (PASEF) to improve ion utilization efficiency and data acquisition speed. The dual TIMS operated the system at 100% duty cycle and recorded the MS/MS mode scanning from 100 to 1700 m/z. The ion mobility was scanned from 0.6 to 1.6 Vs/cm^-2^, and TIMS ion charge control was set to 5e6. The TIMS dimension was calibrated linearly using three selected ions from the Hexakis (1H, 1H, 2H-difluoroethoxy) phosphazene, Hexakis (1H, 1H, 3H-tetrafluoropropoxy) phosphazene Agilent ESI LC/MS tuning mix [m/z, 1/K0: (622.0289, 0.9915 Vs cm^-2^), (922.0097, 1.1996 Vs cm^-2^), (1221,9906, 1.3934 Vs cm^-2^)] in positive mode.

### Data searching, identification, and quantification

The MS/MS data were extracted peak lists under Data Analysis (Bruker Daltonics, Bremen, Germany; version 6.1) and analyzed with Mascot (Matrix Science, London, UK; version 2.7). Mascot was set up to search against the customized protein database described above (customized 5,922 contigs) with a decoy database for false discovery rate (FDR) using digestion enzyme trypsin/Lys-C, parental ion tolerance of 15 ppm and fragment ion mass tolerance of 0.5 Da, respectively. Carbamidomethyl of cysteine (+57.021 Da) was set as the static modification, and oxidation of methionine (+15.995 Da), deamidation of glutamine and asparagine (+0.984 Da), pyro-glutamine formation from N-terminal glutamine (−17.026 Da), as well as phosphorylation of serine, threonine, and tyrosine (+79.966 Da) were specified as the variable modifications. Scaffold Q + S (Proteome Software Inc., Portand, OR, USA; version 5.4.2) was used to validate MS/MS based peptide and protein identifications. Peptide identifications were accepted if they could be established at greater than 95.0% probability by the Peptide Prophet algorithm. Protein quantification was accepted if they could be established at greater than 95.0% probability at significance with at least three peptides with 99.9%. Differences in protein abundance between NP cells and NP small EVs were evaluated using Student’s *t*-test. To be identified as being significantly differentially abundant, proteins should be quantified with at least four unique peptides in both experimental replicates with a *p* < 0.05 and a fold change >1.5 or <0.5.

### Gene ontology analysis, network and clustering of small EV proteins

The gene ontology (GO) and functional enrichment analysis of differentially abundant NP small EV proteins, IVD small EV proteins (NP, AF, and fetal NP), and FAT small EV proteins was performed using the Bioinformatics Resources 6.8 Database for Annotation, Visualization, and Integrated Discovery (DAVID) and the number of genes associated with each term, and enrichment bubble plots were generated using SRplot [116]. Alternatively, ToppFun of the ToppGene Suite was utilized (FDR correction and p-value cut-off 0.05) [117]. Using the search tool for the retrieval of interacting genes/proteins (STRING) database [118], a protein network was built for more abundant and all NP small EV proteins. For medium confidence network construction, default settings were employed. To find clusters in the created protein network, the Markov clustering algorithm (MCL) was utilized and carried out with an inflation parameter of 3. Following clustering, biological processes (BP), molecular functions (MF), and cellular components (CC) clusters were examined in the context of NP small EV protein function. Venny 2.1 (https://bioinfogp.cnb.csic.es/tools/venny/index.html) was used for comparison.

## Results

### Small EV isolation

**Fig 3:**
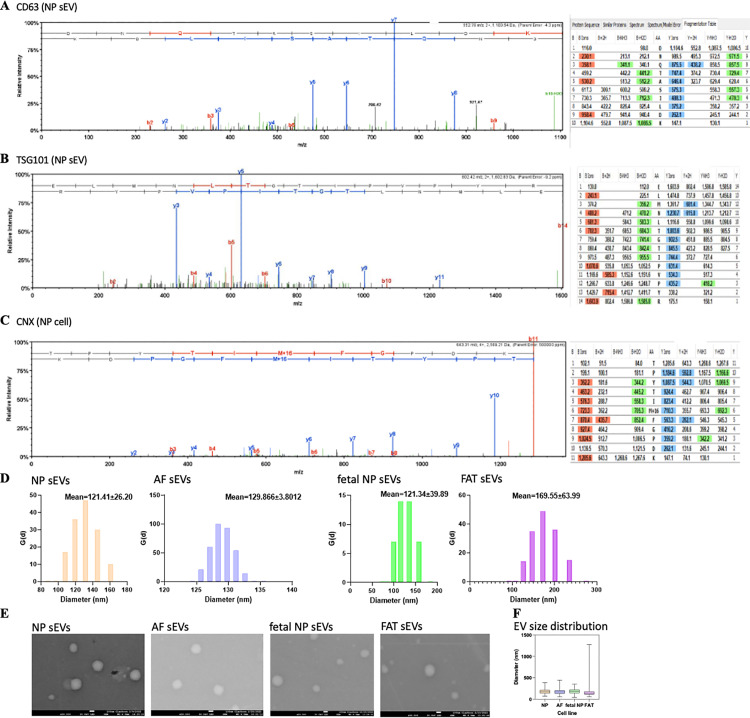
Characterization of small EVs. A) Spectrum for CD63, a cell surface protein of the tetraspanin family and small EV marker. B) Spectrum for tumor susceptibility gene 101 (TSG101), a small EV marker. C) Spectrum for calnexin (CNX), a cell marker. D) DLS data showing the relative amount of particles at each size and the cumulative undersize distribution of small EVs from each parent cell line. E) Scanning electron microscopy (SEM) imaging of small EVs from each parent cell line. The scale bar represents 100 nm. F) Size distribution of DUC purified small EVs determined using ImageJ (n = 150 (NP), n = 157 (AF), n = 130 (FAT), n = 216 (fetal NP)). AF: Annulus fibrosus; DLS: Dynamic light scattering; DUC: Differential ultracentrifugation; EV: Extracellular vesicle; FAT: Adipose tissue; NP: Nucleus pulposus; VB: Vertebral body.

Small EVs were confirmed in the final DUC fraction for all primary bovine parent cell lines through the presence of established small EV markers CD63 and TSG101 and the absence of the cellular marker Calnexin via spectral counts and, if sufficient material was available, by Western blot as shown exemplarily for NP parent cells (Figs 3A–3C and S2). The expected particle size range for small EVs (50–150 nm) was confirmed by DLS (Fig 3D) and ImageJ analysis of SEM micrographs (Fig 3E and 3F). The protein concentration in each small EV fraction was calculated per cultured parent cell (S1 Table).

### Bovine small EV protein database

A bovine small EV protein database was constructed by integrating small EV proteins from ExoCarta [111] and bovine proteins from UniProt [110]. A total of 47,128 bovine protein sequences was downloaded from UniProt, while ExoCarta provided 1,416 bovine and 6,514 human small EV proteins as of October 2, 2023. The BLAST results of human small EV proteins against 47,128 bovine protein sequences resulted in 4,972 bovine proteins, which were merged with bovine small EV proteins obtained from ExoCarta. After sequence clustering with 1,416 bovine small EV proteins and 4,972 bovine proteins homologous to human small EV proteins, the final bovine small EV protein database BOVXCU24 (accessible via PXD056784 and MSV000096081) had 5,922 unique entries.

### Proteome profiling of bovine small EVs

For profiling purposes small EV proteins were first identified in a non-quantitative manner. Small EVs harvested from fetal or adult primary bovine parent cells were compared to existing small EV and MSC small EV data.

#### Bovine small EV proteins in the context of existing data.

**Fig 4:**
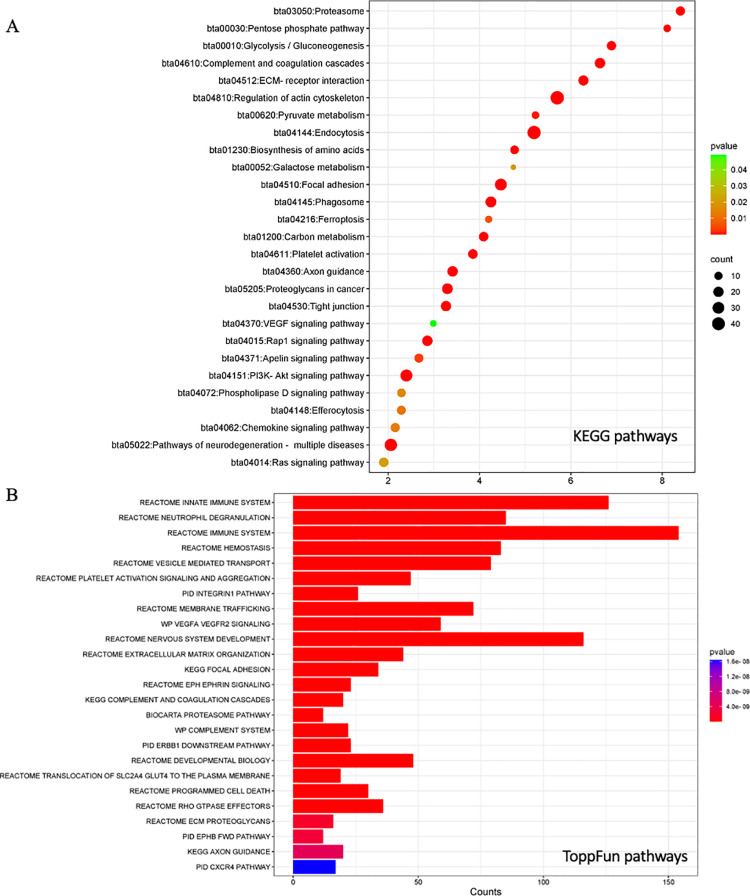
Bovine small EVs and existing data. (A) KEGG pathway analysis in DAVID and (B) ToppFun pathway analysis of 479 shared proteins. DAVID: Database for annotation, visualization, and integrated discovery; KEGG: Kyoto encyclopedia of genes and genomes.

Of the here identified bovine small EV proteins, 479 were shared with ExoCarta [111] or Vesiclepedia [119], both evolving databases for (small)EV research. Our bovine small EV data was essentially consistent with findings by Kugeratski *et al.* identifying Syntenin 1 (syndecan binding protein (SDCBP)) alongside GTPases and membrane proteins such as an integrin subunits and the “classic” tetraspanin small EV markers CD9, CD63 and CD81 [120]. Other established small EV markers such as the programmed cell death 6 interacting protein (PDCD6IP or ALIX) [121] and the tumor susceptibility marker TSG101 known for its role in vacuolar sorting and small EV biogenesis [122] were also detected. Transcripts for these proteins were previously described for the parent cell lines(Tables 1, S2 and S3) [105]. The bovine small EV proteins shared with these small EV databases were associated with a range of biological pathways through functional enrichment analysis in DAVID (Fig 4A) and ToppFun (Fig 4B). Among those, based on fold enrichment over background, the top five highest ranking pathways by fold change (FC) in the KEGG database were proteasome (bta03050), pentose phosphate pathway (PPP) (bta00030), glycolysis/gluconeogenesis (bta00010), complement and coagulation cascades (bta04610), and ECM receptor interaction (bta04512) (Fig 4A). Analysis in ToppFun identified the (innate) immune system (Reactome MM14661), neutrophil degeneration (Reactome M27620), hemostasis (Reactome M8395), nervous system development (M29853), integrin1 (Pathway Interaction Database (PID) M18), and ephrin signaling (Reactome, M27201) amongst the top 200 pathways (Fig 4B, S4 Table).

**Table 1:** Functional enrichment analysis (DAVID) of shared proteins among bovine small EV proteins and the ExoCarta and Vesiclepedia databases.

Type	KEGG Pathway	Pathway ID	Gene Symbol
Metabolism	Glycolysis/ Gluconeogenesis Carbon metabolism Biosynthesis of amino acids Pentose phosphate pathway Pyruvate metabolism Galactose metabolism	bta00010 bta01200 bta01230 bta00030 bta00620 bta00052	ALDOA, ALDOC, ENO1, ENO2, ENO3, FBP1, FBP2, GAPDH, GCK, GPI, HK1, HK2, HK3, LDHA, LDHB, MDH1, MDH2, PCK1, PCK2, PGAM1, PGAM2, PGK1, PGK2, PGM1, PGM2, PKLR, PKM, TPI1
Genetic Information Processing	Proteasome	bta03050	PSMA1, PSMA2, PSMA3, PSMA4, PSMA5, PSMB1, PSMB2, PSMB3, PSMB4, PSMB5, PSMB6, PSMD8, PSMD12, PSMC6
Environmental Information Processing	ECM-receptor interaction PI3K-Akt signaling pathway Ras signaling pathway VEGF signaling pathway	bta04512 bta04151 bta04014 bta04370	COL1A1, COL2A1, COL6A1, COL6A2, COL6A3, FN1, GNB1, GNG12, GNG5, HRAS, ITGA1, ITGA3, ITGA5, ITGA6, ITGB1, ITGB2, ITGB5, ITGB7, ITGAV, KRAS, LAMA5, LAMC1, NRAS, PI3K, RAC1, RHOA, RHEB, VCL, VEGF, YWHAE, YWHAQ, YWHAG
Cellular Processes	Regulation of actin cytoskeleton Endocytosis Focal adhesion Phagosome Tight junction Efferocytosis Ferroptosis	bta04810 bta04144 bta04510 bta04145 bta04530 bta04148 bta04216	ACTN1, ACTR2, ACTR3, ARPC2, ARPC4, ARPC5, ARPC5L, CAPZA2, CAPZB, CDC42, DYNC1H1, EEA1, F2, FGG, FN1, GNG12, HSPA8, HRAS, ILK, ITGA1, ITGA3, ITGA5, ITGA6, ITGB1, ITGB2, ITGB5, ITGB7, ITGAV, LAMP1, LAMP2, M6PR, MSN, NRAS, PFN1, PFN2, RAB10, RAB13, RAB14, RAB35, RAB5B, RAB5C, RAB7A, RAC1, RDX, RHOA, RRAS, RRAS2, SLC16A1, SLC3A2, SMURF1, TKT, TLN1, VAMP3, VASP, VPS35, VPS37B, VPS4A, VPS4B, VPS28
Organismal Systems	Axon guidance Platelet activation Chemokine signaling pathway Complement and coagulation cascades Apelin signaling pathway	bta04360 bta04611 bta04062 bta04610 bta04371	AP2M1, C1QA, C1QC, C3, C5, C6, C7, C8A, C8G, C9, COL1A1, COL3A1, COL6A1, COL6A2, EPHA2, EPHA3, EPHA5, EPHB1, EPHB2, EPHB3, EPHB4, GNAI1, GNAI2, GNAI3, GNAQ, GNG12, GNG5, HRAS, ILK, KRAS, LAMA5, LAMB1, LAMC1, NRAS, PLAU, RAP1B, RDX, RHOA, RAC1, RRAS, RRAS2, THBS1, VASP, VCL, YWHAE
Human Diseases	Pathways of neurodegeneration – multiple diseases Proteoglycans in cancer	bta05022 bta05205	CAPN1, CAPN2, CDC42, COL1A1, COL2A1, COL6A1, COL6A2, COL6A3, DDX5, FLNA, FN1, GNAQ, GNAI1, GNAI2, GNG12, HSPG2, HRAS, KRAS, MMP2, NRAS, PLAU, RAC1, RDX, RRAS, RRAS2, SOD1, THBS1, VDAC1
Signaling and Interaction	Rap1 signaling pathway Phospholipase D signaling pathway	bta04015 bta04072	AGT, DNM2, EPHA2, F2, GNAI1, GNAI2, GNAI3, GNAQ, GNG12, GNG5, HRAS, KRAS, NRAS, PFN1, RAC1, RAP1B, RRAS, RRAS2, RHOA, RHEB, RALA, TLN1, VASP

bta: *Bos taurus;* KEGG: Kyoto Encyclopedia of Genes and Genomes.

#### Small EV proteins from autologous parent cells of different tissues.

**Fig 5:**
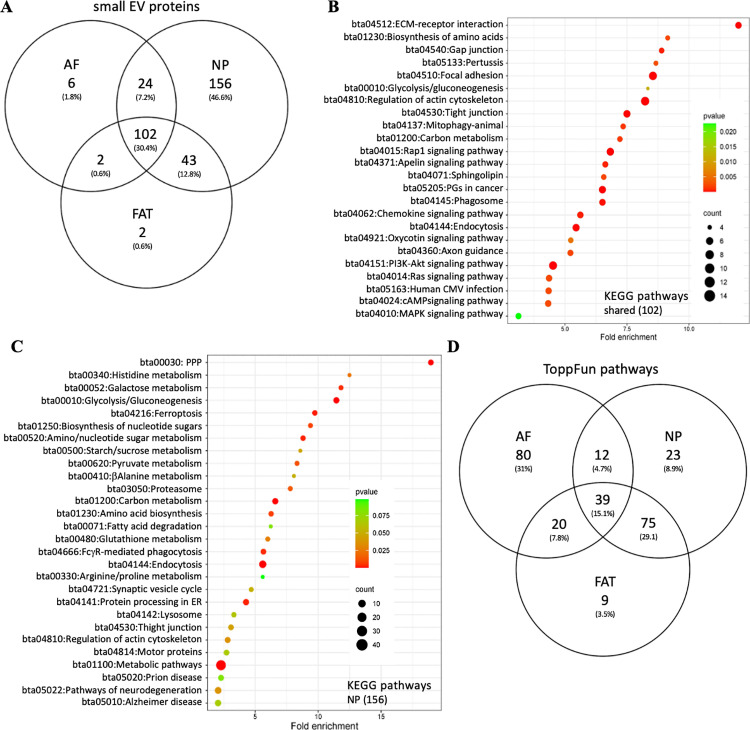
Profiling of small EV proteins from autologous bovine parent cells. A) Venn diagram comparing small EV proteins from autologous NP, AF, and FAT parent cells. B) KEGG pathway analysis in DAVID of 102 shared proteins and (C) KEGG pathway analysis in DAVID of 156 proteins unique to NP small EVs. D) ToppFun pathway analysis of top 200 shared pathways. AF: Annulus fibrosus; DAVID: Database for annotation, visualization, and integrated discovery; FAT: Adipose tissue; KEGG: Kyoto encyclopedia of genes and genomes; NP: Nucleus pulposus.

First, proteome profiling of autologous small EVs isolated from bovine IVD (NP and AF) and adipose (FAT) cells was conducted in an explorative, non-quantitative manner to identify NP parent small EV unique protein signatures. Of the small EV proteins identified for AF, NP and FAT parent cells, a core of 102 (30%) were shared. Functional enrichment analysis in DAVID associated those proteins with a range of important pathways (Fig 5, Table 2). An additional 156 small EV proteins were only identified for NP parent cells. Based on their pathway association in DAVID a metabolic theme stood out, especially the PPP (bta00030) showed a high FC and significance. Associations relevant to NP cell function such as L-ascorbic acid binding (GO:0031418), angiostatin binding (0043532), and general protein stabilization (GO:0050821) were also noted amongst many others (Tables 2 and S5). Functional enrichment analysis in DAVID of the additional 24 small EV proteins shared only between NP and AF parent cells were involved in regulation of the cytoskeleton (bta04810), focal adhesion (bta04510) and endocytosis (bta04144), while additional 43 small EV proteins common only to NP and adipose (FAT) parent cells associated with functions and pathways of the 20S proteasome (KEGG, bta03050). Other small EV proteins in this group were involved with the citrate cycle (bta00020) or calcium ion binding (Figs 5 and S3, Table 2). ToppFun identified 39 pathways common to small EV proteins of all three autologous parent cells, including but not limited to the (innate) immune system, nervous system development, and programmed cell death. Additional 24 were shared only between small EV proteins of NP and AF parent cells and 75 between NP and FAT parent cells (Fig 5D, S6 Table). Among the 23 pathways only associated with small EV proteins from NP parent cells were glycolysis and gluconeogenesis (KEGG, WP) and the PPP (KEGG) represented by glyeraldehyde-3-phosphate dehydrogenase (GAPDH), aldo-keto reductase family member A1 (AKR1A1), phosphofructokinase (PFKL), phosphoglucomutase 1 (PGM1), alcoholdehydrogenase 5 (ADH5), pyruvate kinase (PKLR, PKM), aldehydedehydrogenase 3 and 9 family members (ALDH3B1, ALDH9A1), fructose-bi-phosphate aldolases (ALDOA, ALDOC) glucose-6-phosphate isomerase (GPI), lactate dehydrogenases (LDHA, LDHB), triosephosphate isomerase 1 (TPI1), transketolase (TKT), and glucose-6-phosphate dehydrogenase (G6PD). Also identified was signaling by the Roundabout (ROBO) family (Reactome), transmembrane receptors involved in axon guidance and cell migration through the association with several proteasome 20S subunits, two ribosomal proteins, profilins (PFN1, PFN2), vasodilator stimulated phosphoprotein (VASP), cyclase associated actin cytoskeleton regulatory protein1 (CAP1), RAC family small GTPase 1 (RAC1), protein kinase cAMP-dependent type II regulatory subunit alpha (PRKAR2A), and cell division cycle 42 (CDC42) (S6 Table). Transcripts for these proteins were previously described for the parent cell lines (S3 Table) [105].

**Table 2:** KEGG pathways identified through functional enrichment analysis (DAVID) comparing small EV proteins of autologous bovine NP, AF, and FAT parent cells.

102 shared small EV proteins			
Type	KEGG pathway	ID	Gene symbol
Environmental Information Processing	Regulation of actin cytoskeleton Focal adhesion Rap1 signaling pathway ECM-receptor interaction PI3K-Akt signaling pathway Chemokine signaling pathway Apelin signaling pathway Sphingolipid signaling pathway Ras signaling pathway cAMP signaling pathway MAPK signaling pathway	bta04810 bta04510 bta04015 bta04512 bta04151 bta04062 bta04371 bta04071 bta04014 bta04024 bta04010	ACTN1, C9, COL6A1, COL6A3, FLNA, FN1, GNAI1, GNAI2, GNAI3, GNAQ, GNB1, GSN, HSPA8, HSPB1, HSPG2, ITGA3, ITGAV, ITGB1, LAMC1, MSN, MYH9, PFN1, RAB5B, RAB5C, RAC1, RHOA, RRAS, RRAS2, RAP1B, TLN1, YWHAQ, YWHAE, YWHAG, YWHAZ
Cellular Processes	Tight junction Endocytosis Phagosome Gap junction Mitophagy – animal	bta04530 bta04144 bta04145 bta04540 bta04137	ACTN1, ITGB1, MSN, MYH9, RAB10, RAB13, RAB35, RAB7A, RAB8A, RAB8B, RAC1, RHOA, TUBA1A, TUBB2A
Metabolism	Carbon metabolism Biosynthesis of amino acids Glycolysis/ Gluconeogenesis	bta01200 bta01230 bta00010	ALDOA, GAPDH, PHGDH, PKLR, PKM
Organismal Systems	Axon guidance Oxytocin signaling pathway	bta04360 bta04921	FN1, GNAI1, GNAI2, GNAI3, GNAQ, ITGB1, RAC1, RHOA, RRAS
Human Diseases	Proteoglycans in cancer Human cytomegalovirus infection Pertussis	bta05205 bta05163 bta05133	FLNA, FN1, GNAI1, GNAI2, GNAI3, GNAQ, HSPG2, ITGB1, ITGAV, RAC1, RHOA, RRAS, RRAS2
**156 unique NP small EV proteins**			
**Type**	**KEGG pathway**	**ID**	**Gene symbol**
Metabolism	Metabolic pathways Glycolysis/ Gluconeogenesis Pentose phosphate pathway Carbon metabolism Amino sugar and nucleotide sugar metabolism Galactose metabolism Biosynthesis of amino acids Biosynthesis of nucleotide sugars Pyruvate metabolism Histidine metabolism Glutathione metabolism Starch and sucrose metabolism beta-Alanine metabolism Fatty acid degradation Arginine and proline metabolism	bta01100 bta00010 bta00030 bta01200 bta00520 bta00052 bta01230 bta01250 bta00620 bta00340 bta00480 bta00500 bta00410 bta00071 bta00330	ACP1, ACSL4, ACTN1, ADH5, AHCYL1, ALDH3B1, ALDH9A1, ALDOC, ACO1, ACTN1, ADH5, AHCYL1, ALDH3B1, ALDH9A1, ALDOC, ACO1, ANXA6, ATIC, ATP5F1A, ATP5F1B, ATP6AP1, CDC42, CHMP4B, CHMP6, CNDP2, DNM2, EEF1A1, FASN, GAA, GALK1, GANAB, G6PD, GSN, GPI, GRHPR, LAP3, LDHB, MAN1A1, MTHFD1, MOGS, NAGK, PLOD1, PLOD3, PFKL, PGM1, PAFAH1B2, PRDX6, PRXL2B, PSAT1, RAB22A, RENBP, TKT, TSG101, VPS35, VPS36, VPS4B, VTA1
Genetic Information Processing	Protein processing in endoplasmic reticulum Proteasome	bta04141 bta03050	APRT, CAPN1, CKAP4, CRYAB, DNAJA1, GANAB, MAN1A1, MOGS, PRXL2B, PSMD8, PSMD12, PSMC6, PSMB3, SEC13
Environmental Information Processing	Regulation of actin cytoskeleton Endocytosis Fc gamma R-mediated phagocytosis	bta04810 bta04144 bta04666	AP2M1, ARPC2, ARPC4, ARPC5L, CDC42, CHMP4B, CHMP6, DNM2, NCKAP1, PFN2, VASP, VPS35
Cellular Processes	Ferroptosis Tight junction Synaptic vesicle cycle Lysosome Motor proteins	bta04216 bta04530 bta04721 bta04142 bta04814	AP2M1, ATP6AP1, ATP6V0E2, CAPZA2, CDK5, DCTN2, DNM2, FUCA1, LAMP2, MYH14, MYO1B, NAPA, TPP1, TUBB6
Human Diseases	Pathways of neurodegeneration – multiple diseases Alzheimer disease Prion disease	bta05022 bta05010 bta05020	ATP5F1A, ATP5F1B, CAPN1, CDK5, DCTN2, PSMB3, PSMD12, PSMD8, PSMC6, TUBB6, UBA1
**24 proteins shared between NP and AF small EVs**			
**Type**	**KEGG pathway**	**ID**	**Gene symbol**
Cellular Processes	Regulation of actin cytoskeleton Endocytosis Focal adhesion	bta04810 bta04144 bta04510	ACTR2, CAPZB, CHMP2A, FLNB, HRAS, ITGA1, IQGAP1, IQGAP2, RALA, RDX
Human Diseases	Proteoglycans in cancer Salmonella infection	bta05205 bta05132	ACTR2, CHMP2A, FLNB, HRAS, IQGAP1, IQGAP2, ITGA1, RALA, RDX
**43 proteins shared between NP and FAT small EVs**			
**Type**	**KEGG pathway**	**ID**	**Gene symbol**
Human Diseases	Amyotrophic lateral sclerosis Spinocerebellar ataxia Prion disease Huntington disease Pathways of neurodegeneration – multiple diseases Parkinson disease Alzheimer disease Salmonella infection	bta05014 bta05017 bta05020 bta05016 bta05022 bta05012 bta05010 bta05132	ACLY, ACTR1A, ACTR3, ANXA7, ANXA11, ATP6V1B2, C5, CYFIP2, IDH1, M6PR, PSMA1, PSMA2, PSMA3, PSMA4, PSMA5, PSMB2, PSMB5, PSMB6, THBS1, VAMP3, VCL
Cellular Processes	Proteasome Regulation of actin cytoskeleton Phagosome	bta03050 bta04810 bta04145	ACLY, ACTR1A, ACTR3, ATP6V1B2, C5, CYFIP2, IDH1, M6PR, PSMA1, PSMA2, PSMA3, PSMA4, PSMA5, PSMB2, PSMB5, PSMB6, THBS1, VAMP3, VCL
Metabolic Pathways	Citrate cycle (TCA cycle)	bta00020	ACLY, IDH1

AF: Annulus fibrosus; bta: *Bos taurus*; cAMP: Cyclic adenosine monophosphate; DAVID: Database for annotation, visualization, and integrated discovery; ECM: Extracellular matrix; FAT: Subcutaneous adipose tissue; KEGG: Kyoto encyclopedia of genes and genomes; MAPK: Mitogen-activated protein kinase; NP: Nucleus pulposus.

#### Small EV proteins from adult or fetal NP and MSC parent cells.

**Fig 6:**
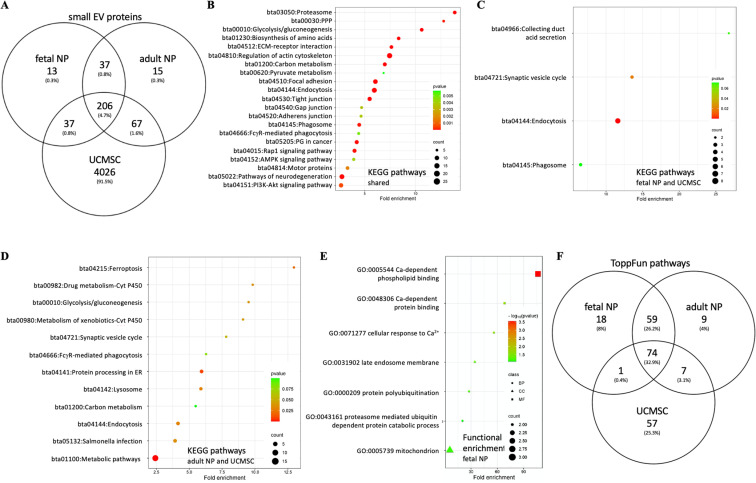
Nucleus pulposus and MSC small EV profiling. A) Comparison of small EV proteins from fetal and adult NP parent cells to those of UCMSCs (128). B) KEGG pathway analysis in DAVID of 206 shared proteins (C) KEGG pathway analysis in DAVID of small EV proteins shared by fetal NP and UCMSC parent cells. D) KEGG pathways analysis in DAVID of small EV proteins shared between adult NP and UCMSC parent cells. E) Functional enrichment analysis in DAVID of 13 proteins only identified for fetal NP small EVs. F) ToppFun pathway analysis of top 200 shared pathways. DAVID: Database for annotation, visualization, and integrated discovery; KEGG: Kyoto encyclopedia of genes and genomes; NP: Nucleus pulposus; UCMSCs: Umbilical cord mesenchymal stem cells.

A comparison of the small EV protein profile of adult and fetal NP parent cells with that of MSC small EVs was conducted to investigate if NP small EV proteins can positively impact on cell homeostasis or progenitor cell mobilization. In this study, data from human umbilical cord mesenchymal stem cells (UCMSCs) was chosen for comparison as it was the most comprehensive MSC small EV protein dataset at the time of the analysis [114]. Of the small EV proteins identified, 206 were shared between fetal NP, adult NP, and UCMSC parent cells; additional 37 only between fetal and adult NP parent cells; 67 only between adult NP and UCMSC parent cells; and 37 only between fetal NP and UCMSC parent cells. Another 13 and 15 were only identified in small EVs of fetal or adult NP cells, respectively (Fig 6A). Functional enrichment analysis by DAVID of the 206 shared small EV proteins identified proteasome (bta03050), PPP (bta00030) and glycolysis/gluconeogenesis (bta00010) as pathways with high significance and highest FC (Figs 6B and S4, Table 3). DAVID analysis associated the 37 small EV proteins identified for fetal NP and UCMSC parent cells essentially with endocytosis and vesicle formation, while small EV proteins shared between UCMSC and adult NP parent cells related to metabolism, though the FC was low (Fig 6C–6E, Tables 3 and S7). ToppFun pathway analysis for each of the three parent cell lines identified a total of 74 pathways common to all three sources, including the platelet-derived growth factor receptor β (PDGFRB) pathway (PID), and β-catenin independent WNT signaling (Reactome) (Fig 6F, S8 Table). Among pathways linked to small EV proteins from UCMSC and adult NP parent cells were signaling by ROBO receptors (Reactome) and for UCMSC and fetal NP parent cells signal transduction by growth factor receptors and second messengers (Reactome). Many of the 59 pathways associated with small EV proteins shared between adult and fetal NP parent cells affected various signaling pathways including Notch signaling, but often through their proteasome association. Glycolysis/gluconeogenesis (KEGG) and aerobic glycolysis (WP) was identified, essentially via most proteins mentioned for glycolysis above (Tables 3, S7 and S8). Hematopoietic stem cells are amongst the best studied progenitor cells and can be mobilized through the G-protein-coupled chemokine receptor CXCR4 and its ligand CXCL12 [123]. These proteins were not detected in small EVs here, though transcripts for both were previously identified in NP cell lines with 5.9 and 1.8 transcripts per million (TPM), respectively [105]; and an association with the CXCR4 pathway (PID, M124) was suggested for small EV proteins shared between adult and fetal NP parent cells based on the identification of 14 proteins containing several integrin and G-protein subunits, receptor for activated C kinase (RACK1), RAC1, vacuolar protein sorting 4 homolog B (VPS4B), RAS homolog family member A (RHOA) and CDC42. Transcripts for all were previously identified in NP cell lines [105].

**Table 3:** KEGG pathways identified through functional enrichment analysis (DAVID) comparing small EV proteins of adult and fetal NP cells with UCMSC cells.

206 shared small EV proteins			
Type	KEGG Pathway	ID	Gene symbol
Genetic Information Processing	Proteasome	bta03050	PSMB6, PSMA5, PSMD8, PSMD12, PSMA3, PSMA4, PSMB5, PSMC6, PSMB2, PSMA1, PSMA2
Metabolism	Glycolysis/Gluconeogenesis Carbon metabolism Biosynthesis of amino acids Pentose phosphate pathway Pyruvate metabolism	bta00010 bta01200 bta01230 bta00030 bta00620	ACO1, ALDH9A1, ALDOA, GAPDH, G6PD, GPI, IDH1, LDHA, LDHB, PFKL, PGM1, PGD, PHGDH, PKM, PKLR, PSAT1, TPI1
Environmental Information Processing	Endocytosis Tight junction Rap1 signaling pathway PI3K-Akt signaling pathway AMPK signaling pathway	bta04144 bta04530 bta04015 bta04151 bta04152	ACTN1, ACTR2, ACTR3, ARF1, ARPC2, ARPC4, CAPZA2, CAPZB, CDC42, CHMP2A, CHMP4B, COL6A1, COL6A3, EHD2, EEF2, FASN, FN1, GNAI1, GNAI2, GNAI3, GNB1, IQGAP1, ITGA1, ITGA3, ITGAV, ITGB1, LAMA5, LAMC1, MSN, MYH9, MYH14, PDCD6IP, PFN1, PFKL, RAB2A, RAB5B, RAB5C, RAB7A, RAB8A, RAB8B, RAB10, RAB13, RAB14, RAB35, RALA, RAP1B, RDX, RHEB, RHOA, RRAS, RRAS2, TSG101, THBS1, TLN1, VPS35, VPS4B, YWHAQ, YWHAE, YWHAZ
Cellular Processes	Focal adhesion ECM-receptor interaction Gap junction Phagosome Platelet activation Regulation of actin cytoskeleton Motor proteins Cytoskeleton in muscle cells	bta04510 bta04512 bta04540 bta04145 bta04611 bta04810 bta04814 bta04820	ACTN1, ACTR2, ACTR3, ARPC2, ARPC4, BGN, CAPZA2, CAPZB, CDC42, COL6A1, COL6A3, FLNA, FLNB, FN1, GSN, ITGA1, ITGA3, ITGAV, ITGB1, LAMA5, LAMC1, MYH14, MYH9, NID1, RHOA, TTN, VCL
Human Diseases	Alzheimer disease Parkinson disease Huntington disease Spinocerebellar ataxia Prion disease Pathways of neurodegeneration – multiple diseases Proteoglycans in cancer	bta05010 bta05012 bta05016 bta05017 bta05020 bta05022 bta05205	ACTR1A, APOE, ATP5F1A, ATP5F1B, CAPN1, CAPN2, C5, FLNA, FN1, GNAQ, GNAI1, GNAI2, GNAI3, HSPA5, HSPG2, IQGAP1, ITGAV, ITGB1, LAMC1, MSN, PSMC6, PSMA1, PSMA2, PSMA3, PSMA4, PSMA5, PSMB2, PSMB5, PSMB6, PSMD12, PSMD8, RAC1, RDX, RRAS, RRAS2, THBS1, TUBB2A, TUBB6
Organismal Systems	Fc gamma R-mediated phagocytosis	bta04666	ACTR2, ACTR3, ARPC2, ARPC4, CDC42, GSN, RAC1
**37 proteins shared by adult and fetal NP small EVs**			
**Type**	**KEGG Pathway**	**ID**	**Gene symbol**
Human diseases	Viral carcinogenesis Alcoholism Salmonella infection	bta05203 bta05034 bta05132	ARL8A, H2BC18, H4C4, HRAS, TUBA1A, YWHAH
**67 proteins shared by adult NP and UCMSC small EVs**			
**Type**	**KEGG Pathway**	**ID**	**Gene symbol**
Metabolism	Metabolic pathways Carbon metabolism Glycolysis/Gluconeogenesis	bta01100 bta01200 bta00010	ACP1, ACSL4, ADH5, ALDOC, ATP6V1B2, GAA, GALK1, GANAB, GRHPR, GSTP1, LAP3, MTHFD1, MOGS, NAGK, PAFAH1B2, PLOD1, PLOD3, TKT
Genetic Information Processing	Protein processing in endoplasmic reticulum	bta04141	CKAP4, DNAJA1, GANAB, MOGS, SEC13
Cellular Processes	Ferroptosis Lysosome Endocytosis Fc gamma R-mediated phagocytosis	bta04216 bta04142 bta04144 bta04666	ACSL4, AP2M1, ARPC5L, CP, DNAJA1, DNM2, FUCA1, GAA, LAMP2, PCBP1, RAB22A, TPP1, VASP, VPS36
Human Diseases	Salmonella infection	bta05132	ARPC5L, DCTN2, DNM2, PFN2, SNX18
Nervous System	Synaptic vesicle cycle	bta04721	AP2M1, ATP6V1B2, DNM2
**37 proteins shared by fetal NP and UCMSC small EVs**			
**Type**	**KEGG Pathway**	**ID**	**Gene symbol**
Environmental Information Processing	Endocytosis	bta04144	ARPC5, CHMP1B, EEA1, EHD4, IST1, SNX12, SNX3, VPS28
Cellular Processes	Phagosome	bta04145	ATP6V0A1, ATP6V1D, EEA1
Organismal Systems	Synaptic vesicle cycle Collecting duct acid secretion	bta04721 bta04966	ATP6V0A1, ATP6V1D, STXBP1
**13 small EV proteins unique to fetal NP cells**			
**Category/type**	**GO Term/pathway**	**ID**	**Gene Symbol**
BF/ Cellular Processes and Protein Processing	cellular response to calcium ion protein polyubiquitination proteasome-mediated ubiquitin-dependent protein catabolic process	GO:0071277 GO:0000209 GO:0043161	CPNE3, CPNE5, SMURF1, SOD1
MF/ Calcium Binding	calcium-dependent phospholipid binding calcium-dependent protein binding	GO:0005544 GO:0048306	ANXA13, CPNE3, CPNE5, S100A14
CC/ Intracellular Membrane bounded organelle	late endosome membrane mitochondrion	GO:0031902 GO:0005739	CHMP2B, CPNE3, GPX4, SOD1, VAMP8
KEGG/ Cellular Processes	Endocytosis	bta04144	SMURF1, CHMP2B

BF: Biological function; bta: *Bos taurus*; CC: Cellular components; DAVID: Database for annotation, visualization, and integrated discovery; FAT: Adipose tissue; KEGG: Kyoto encyclopedia of genes and genomes; NP: Nucleus pulposus; MF: Molecular function; UCMSC: Umbilical cord mesenchymal stem cells.

### Comparative analysis of the NP small EV and parent proteome

**Fig 7:**
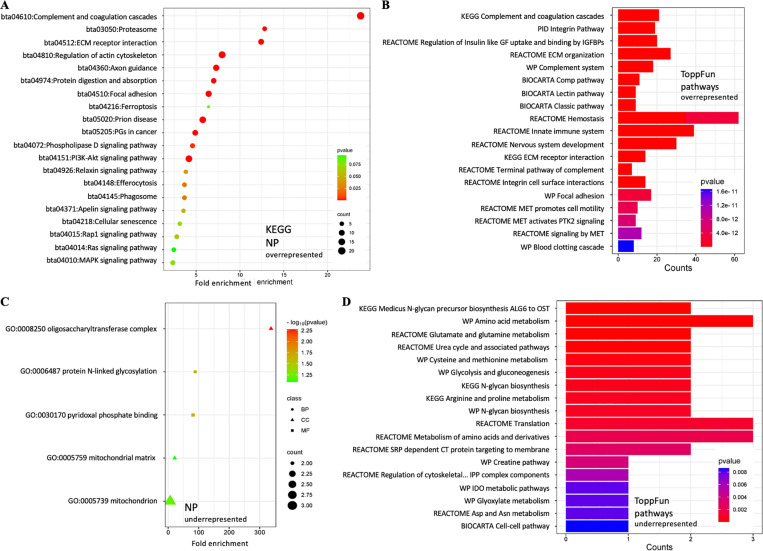
Quantitative functional enrichment analysis of NP small EV proteins. A) KEGG pathway analysis and (B) ToppFun pathway analysis of 141 proteins more abundant in NP small EVs as compared to their parent cells. C) Functional enrichment analysis and (D) ToppFun pathway analysis of ten proteins reduced in NP small EVs as compared to their parent cells. DAVID: Database for annotation, visualization, and integrated discovery; KEGG: Kyoto encyclopedia of genes and genomes; NP: Nucleus pulposus.

**Fig 8:**
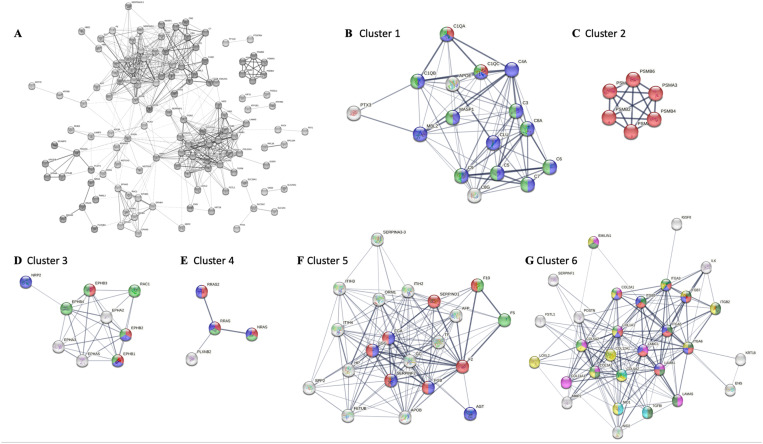
Functional clusters of more abundant NP small EV proteins. A) STRING network of 141 more abundant NP small EV proteins; B) Cluster 1: Complement component C1q complex (red), complement and coagulation cascades (blue), complement activation classical pathway (green); C) Cluster 2: Proteasome (red); D) Cluster 3: Axon (blue), axon guidance receptor activity (red), Ephrin signaling (green); E) Cluster 4: Ras signaling (red), axon guidance (green) and MAPK signaling pathway (blue); F) Cluster 5: Hemostasis (green), regulation of MAPK cascade (blue), complement and coagulation cascades (red); G) Cluster 6: ECM (pink), integrin binding (light green), collagen binding (sky blue), ECM receptor interaction (red), PI3K/AKT signaling pathway (blue), ECM organization (yellow), signaling by RTKs (dark green). STRING: Search tool for the retrieval of interacting genes/proteins; NP: Nucleus pulposus.

Bioactive molecules in the membrane or lumen of small EVs might end up there by chance, reflecting their abundance in the parent cell, or they could be deposited/ removed during small EV biogenesis. To identify differentially abundant small EV proteins, quantitative proteomics of NP small EVs and their respective parent cells was conducted. We identified a total of 151 differentially abundant proteins, which were subjected to functional enrichment analysis using DAVID and ToppFun (Figs 7 and S5, Tables 4, S9 and S10), then protein clusters were visualized in STRING (Fig 8).

**Table 4:** KEGG pathways identified through functional enrichment analysis (DAVID) after quantitative comparison of NP small EV and parent cell proteins.

141 proteins more abundant in NP small EVs			
Type	KEGG pathway	ID	Gene symbol
Genetic Information Processing	Proteasome	bta03050	PSMA3, PSMA4, PSMB1, PSMB2, PSMB4, PSMB6
Environmental Information Processing	PI3K-Akt signaling pathway Phospholipase D signaling pathway Rap1 signaling pathway MAPK signaling pathway Ras signaling pathway	bta04151 bta04072 bta04015 bta04010 bta04014	AGT, CACNA2D1, COL1A1, COL2A1, COL6A2, EPHA2, EPHA3, EPHA5, EPHB1, EPHB2, EPHB3, EPHB4, F2, ILK, ITGA3, ITGA5, ITGA6, ITGB2, ITGB5, ITGB7, KRAS, LAMA5, LAMB1, LAMC1, NRAS, RAC1, RHEB, RRAS, RRAS2
Cellular Processes	Cellular senescence Apelin signaling pathway Axon guidance Relaxin signaling pathway Focal adhesion ECM-receptor interaction Regulation of actin cytoskeleton Efferocytosis Phagosome Ferroptosis	bta04218 bta04371 bta04360 bta04926 bta04510 bta04512 bta04810 bta04148 bta04145 bta04216	ADAM10, CACNA2D1, C1QA, C1QC, C3, C5, C6, C7, C8A, C8G, C9, COL1A1, COL2A1, COL3A1, COL6A2, EPHA2, EPHA3, EPHA5, EPHB1, EPHB2, EPHB3, EPHB4, F2, FTH1, ILK, ITGA3, ITGA5, ITGA6, ITGB2, ITGB5, ITGB7, KRAS, LAMA5, LAMB1, LAMC1, LAMP2, MMP2, NOTCH3, NRAS, PLAU, PLXNB2, RAC1, RRAS, RRAS2, SLC3A2, SLC16A1, TF
Organismal Systems	Complement and coagulation cascades Protein digestion and absorption	bta04610 bta04974	ATP1B1, C1QA, C1QC, C3, C5, C6, C7, C8A, C8G, C9, CLU, COL11A1, COL12A1, COL1A1, COL2A1, COL3A1, COL5A2, COL6A2, F10, F2, F5, FGA, FGB, FGG, ITGB2, LOC107131209, MASP1, PSMB1, PSMB2, PSMB4, PSMB6, PSMA3, PSMA4, RAC1, SERPIND1, SERPINF2, SLC3A2
Human Diseases	Proteoglycans in cancer Prion disease	bta05171 bta05020	C1QA, C1QC, C5, C6, C7, C8A, C8G, C9, COL1A1, COL2A1, ITGA5, ITGB2, ITGB5, KRAS, LAMC1, MMP2, NRAS, PLAU, PSMB1, PSMB2, PSMB4, PSMB6, PSMA3, PSMA4, RAC1, RRAS, RRAS2
**10 proteins less abundant in NP small EVs**			
**Category/type**	**GO Term/pathway**	**ID**	**Gene Symbol**
BF – Metabolic Processes	protein N-linked glycosylation	GO:0006487	RPN2, DAD1
MF – Binding	pyridoxal phosphate binding	GO:0030170	OAT, GOT2
CC – Intracellular Membrane bounded organelle	oligosaccharyltransferase complex mitochondrion mitochondrial matrix	GO:0008250 GO:0005739 GO:0005759	DAD1, GOT2, LDHAL6B, OAT, RPN2, SLC25A3
KEGG – Metabolism	Metabolic pathways Various types of N-glycan biosynthesis Arginine and proline metabolism Cysteine and methionine metabolism N-Glycan biosynthesis	bta01100 bta00513 bta00330 bta00270 bta00510	DAD1, GOT2, LDHAL6B, OAT, RPN2

BF: Biological function; bta: *Bos taurus*; CC: Cellular components; DAVID: Database for annotation, visualization, and integrated discovery; FAT: Adipose tissue; KEGG: Kyoto encyclopedia of genes and genomes; NP: Nucleus pulposus; MF: Molecular function; UCMSC: Umbilical cord mesenchymal stem cells.

#### Differentially abundant NP small EV proteins.

Of the 151 proteins identified as differentially abundant, 141 proteins were more abundant in NP small EVs and ten less abundant relative to their parent cells (Tables 4, S9 and S10). Amongst the more abundant proteins were Syntenin 1 (SDCBP), integrin subunit β1 (ITGB1) and many GTPases, consistent with the small EV core proteome [120]. Pathways were associated with important cell signaling cascades as well as cell and niche homeostasis as described in more detail below (Figs 7 and S5). While no pathways were identified for the ten less abundant proteins using DAVID, these proteins were involved with posttranslational modifications such as oligosaccharyltransferase (GO:0008250) and N-linked glycosylation (GO:0006487), and as coenzyme in the biosynthesis of amino acids, neurotransmitters and sphingolipids via pyridoxal phosphate (PLP) binding (GO:0030170). They further associated with mitochondria (GO:0005739) and mitochondria matrix (GO:0005759) (Fig 7C, Tables 4 and S9). Functional enrichment using ToppFun identified amino acid metabolism (WP, M39570), transaminase activity (GO:0008483) and glutamate and glutamine metabolism (Reactome, M27851) as associated with underrepresented NP small EV proteins through an association with glutamic-oxaloacetic transaminase 2 (GOT2), tryptophanyl-tRNA sythetase 1 (WARS1) and ornithine aminotransferase (OAT). The mitochondrial lactate dehydrogenase A like 6B (LDHAL6B) was also underrepresented (Fig 7D, S9 and S10 Tables). Transcripts for all were previously detected in NP cells, *LDHAL6B* at very low levels though (S3 Table) [105].

#### More abundant NP small EV proteins with immunomodulatory and housekeeping functions.

STRING analysis of the 141 more abundant NP small EV proteins revealed at least six distinct clusters (Fig 8A). The healthy NP is considered immune privileged [124], yet important elements in the innate immune response were identified in cluster 1 as relevant for complement activation and opsonization, including proteins associated with the complement component C1Q complex (GO:0062167), complement and coagulation cascades (bta04610) and complement activation classical pathway (WP977). The cluster contained most members of the complement cascade (C1 to C9) alongside the opsonins Pentraxin 3 (PTX3) and mannose binding lectin 2 (MBL2); the complement activating component mannose-binding lectin-associated serine protease 1 (MASP1); and Apolipoprotein E (APOE), an inhibitor of the classic complement cascade via C1Q binding [125] (Fig 8B). Transcripts for all proteins except MBL2 including various members of the complement complex, except C1Q and C4, were previously identified for NP cells, though typically with very low TPM (S3 Table) [105]. Notably, no interleukins were identified despite their transcription in NP parent cells (S2 and S3 Tables) [105]. Proteasomes, intracellular non-lysosomal protein degradation complexes, as seen in cluster 2 have important functions in cell homeostasis, physiology and development [126]. This cluster was comprised of subunits of the standard 20S proteasome, namely α3, α4, β1,β2, β4, and β6 (Fig 8C, Table 4). Transcripts for all proteasome units were previously identified for NP cells [105].

#### More abundant NP small EV proteins participate in cell signaling.

Small EV proteins can be associated with the small EV membrane as ion channels, transporters, receptors, or internal cargo. Of the 141 more abundant NP small EV proteins 38% were membrane associated. Amongst those were seven receptor tyrosine kinases (RTK), all of the EPH receptor type, two Notch receptors (NOTCH 2 and 3), one chloride anion channel (Tweety homolog 3 (TTYH3)), the auxiliary subunit of a calcium-voltage gated channel (CACNA2D1), and the insulin-like growth factor 2 receptor (IGF2R). All proteins have been previously identified as small EV associated and transcripts for all were previously identified for NP cells (Tables 4 and S3) [105,111].

***EPH receptor tyrosine kinase (RKT) – RAS/MAPK signaling axis:*** Cluster 3 was composed of nine NP small EV proteins (Fig 8D). Of those the Ephrin receptors EPHB1, EPHB2, EPHB3, EPHB4 and the RAC family small GTPase 1 (RAC1), a plasma membrane associated small GTPase involved in a range of cellular events [127–129], associated with the term ephrin signaling (bta3928664). EPHB1, EPHB2 and EPHB3 additionally associated with the term axon guidance receptor activity (GO:0008046), and EPHB1, EPHB2 and Neuropilin 2 (NRP2), a transmembrane protein and high-affinity receptor for some semaphorins [130], with the term axon (GO:0030424). Several components of the RAS pathway signaling cascade were also enriched in NP small EVs, forming the small cluster 4. Part of this cluster were RAS proto-oncogenes and GTPases NRAS, RRAS and RRAS2, also PlexinB2 (PLXNB2), a cell surface receptor for semaphorins, associating with the RAS (bta04014) and MAPK (bta04010) signaling pathways as well as axon guidance (bta04360) (Fig 8E). Cluster 5 consisted of 20 NP small EV proteins associated with the regulation of MAPK cascade (GO:0043408), namely the fibrinogen α (FGA), β (FGB) and γ(FGG) chains and angiotensinogen (AGT) (Fig 8F). Transcripts for all proteins but EPHB1, FGA and AGT were previously identified for NP cells, though some with low TPM (S3 Table). Interestingly, transcripts for ephrin ligands *(EFNA1–5* and *ENFNB1–3*) were previously detected in NP cells, however, the ligands were not enriched in NP small EVs (S2 and S3 Tables) [105].

***ECM-integrin signaling axis:*** The largest cluster 6 contained 29 more abundant NP small EV proteins associated with ECM structural constituents (GO:0005201), ECM organization (GO:0030198) and ECM receptor interaction (bta04512), integrin- (GO:00005178) and collagen- (GO:00005518) binding. Amongst the proteins in cluster 6 were collagens (COL) 1, 2, 3, 5, 6, 11 and 12, various integrin subunits (ITG), laminins (LAMA5, LAMB1, LAMC1), and Keratin 18 (KRT18) an intermediate filament. Also, Matrix metalloproteinase 2 (MMP2), Transforming growth factor βinduced (TGFβI) and more (Fig 8G). Especially the laminins and integrins but also COL1 and COL2 were associated with PI3K/AKT signaling (bta04151) through RTK signaling (bta9006934) and ECM receptor interaction (Fig 8G). Transcripts for all but KRT18 were previously identified for our NP cells (S3 Table) [105].

### NP small EV proteins with links to major cellular events

**Fig 9:**
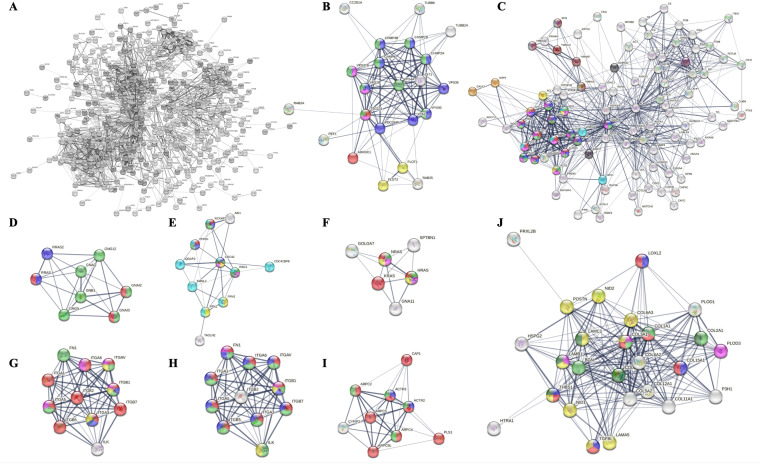
Functional clusters of all identified NP small EV proteins. A) STRING network of 484 NP small EV proteins; B) Cluster 1: Extracellular vesicle biogenesis (red), multivesicular body sorting pathway (blue), ESCRT I complex (pink), ESCRT (green), Flotillin complex (yellow); C) Cluster 2: Detection of oxidative stress (black), pentose phosphate pathway (red), glycolysis/gluconeogenesis (blue), biosynthesis of amino acids (green), carbon metabolism (pink), pyruvate metabolism (dark green), TCA cycle (yellow), glutathione metabolism (sky blue), HIF1 signaling pathway (purple), amino sugar and nucleotide sugar metabolism (ochre yellow), activation of BAD and translocation to mitochondria (brown), regulation of localization of FOXO transcription factors (grey); D) Cluster 3: Regulation of Schwann cell migration (blue), G-protein coupled receptor signaling pathway (green), axon guidance (red); E) Cluster 4: Rac protein signal transduction (red), RAS protein signal transduction (blue), small GTPase mediated signal transduction (green), RAP1 signaling pathway (yellow), EPHB-mediated forward signaling (pink), VEGFA/VEGFR2 pathway (dark green), signaling by Rho GTPases (sky blue); F); Cluster 5: MAP2K and MAPK activation (red), RAS signaling pathway (green), MAPK signaling pathway (pink), PI3K/AKT signaling pathway (yellow); G) Cluster 6 version 1: Cell adhesion mediated by integrin (red), mesodermal cell differentiation (blue), angiogenesis (green), regulation of small GTPase mediated signal transduction (yellow), negative regulation of apoptotic process (pink); H) Cluster 6 version 2: ECM receptor interaction (red), focal adhesion (green), PI3K/AKT signaling pathway (blue), axon guidance (yellow), TGFβ signaling pathway (pink); I) Cluster 7: Positive regulation of lamellipodium assembly (blue), actin cytoskeleton organization (red), EPHB mediated forward signaling (green); J) Cluster 8: ECM assembly (pink), cartilage development (green), angiogenesis (blue), blood vessel development (red), cell adhesion (yellow), PI3K/AKT signaling pathway (dark green). ECM: Extracellular matrix; ESCRT I: Endosomal sorting complex required for transport I; STRING: Search tool for the retrieval of interacting genes/proteins; NP: Nucleus pulposus.

STRING was also used to identify NP small EV protein clusters (Fig 9A) based on all confirmed 484 NP small EV proteins identified here, not limited to those differentially abundant in NP small EVs over their parent cells described above (S2 Table). The cluster of 22 proteins in Fig 9B, identified proteins involved in small EV biogenesis and the endosomal sorting complex (ESCRT). The largest cluster in Fig 9C contained 87 NP small EV proteins and was dominated by carbon metabolism (bta01200) with associations to metabolic processes including but not limited to core processes in energy production, with GAPDH taking on a central role (Fig 9C). Also, part of this cluster were proteins involved in oxidative stress associated hypoxia inducible factors (HIF) signaling (bta04066), glutathione metabolism (bta00480), and proteins associated with pathways downstream of PI3K/AKT signaling (R-bta-111447, R-bta-9614399). A cluster of eight proteins involved in GPCR signal transduction in Fig 9D showed ties to axon guidance and Schwann cell migration. The cluster of 11 proteins in Fig 9E associated with EPHB-mediated forward signaling (R-bta-3928662) and connected to other important signaling pathways such as signaling by RHO GTPases (R-bta-194315) and VEGFA/VEGFR2 (R-bta-4420097). The small cluster of six proteins in Fig 9F related to the RAS proto-oncogenes which are crucial in many signal transduction pathways including the RAS (bta04014), PI3K/AKT (bta04151) and MAPK (bta04010,) signaling pathway and related MAP2K and MAPK activation (R-bta-5674135). The cluster of 11 proteins in Fig 9G–9H was largely composed of different integrin subunits, fibronectin (FN1) and the integrin-linked kinase (ILK), all involved in a range of biological processes including cell adhesion (GO:0033627), mesodermal cell differentiation (GO:0048333), angiogenesis (GO:0001525), regulation of small GTPase mediated signal transduction (GO:0051056), negative regulation of apoptotic process (GO:0043066) and ties to ECM receptor interaction (bta04512), focal adhesion (bta04510), TGFβ signaling (WP1045), the PI3K/AKT signaling pathway and axon guidance (bta04360). The cluster of nine proteins seen in Fig 9I essentially associated with biological processes such as actin filament polymerization (GO:0030041), actin cytoskeleton organization (GO:0030036,) and positive regulation of lamellipodium (GO0010592), hence affiliated with cell migration. The cluster of 26 NP small EV proteins shown in Fig 9J associated with the PI3K/AKT signaling pathway affecting a range of biological processes including ECM assembly (GO:0085029), cartilage development (GO:0051216), angiogenesis (GO:0001525), blood vessel development (GO:0001568) and cell adhesion (GO:0007155).

### Considerations regarding a serum corona

Recent work demonstrated that plasma proteins spontaneously form a corona around the surface of EVs or virus particles [131]. Many cells require fetal bovine serum (FBS) during *in vitro* culture. Of corona proteins reported for the serum of healthy participants [131], 11 were identified in our NP small EV protein data set, namely Clusterin (CLU); fibrinogen chains α, β and γ (FGA, FGB, FGG); complements C3 and C4; α1-acid protein 1 (ORM1); Ceruloplasmin (CP) involved in iron oxidation without the production of oxygen radicals thus preventing tissue damage; Inter-alpha-trypsin inhibitor heavy chains ITIH2, ITIH4; and Apolipoprotein E (APOE); with *CLU*, *APOE* and *CP* showing high (TPM > 2000) and moderate transcript levels (TPM 137 and 45) in NP cells, respectively. Amongst the three autologous parent cell types *CLU* transcript levels were previously found highest in NP cells. No transcripts were identified in NP, AF or FAT parent cell for *ITIH2*, *FGA* and *C4,* while only very low levels were detected for *ITIH4* and *ORM1*. Amongst proteins found in viral coronas [131], APOB, APOE, C3 and C4 overlapped with our data set of NP small EV proteins, with *C3* and *APOB* being low level or not transcribed, respectively (S3 Table) [105]. Via ToppFun we identified associations with pathways like neutrophil degranulation, innate immune system, immune system, platelet activation signaling and aggregation, nervous system, VEGFA/VEGFR2 signaling, focal adhesion and regulation of the actin cytoskeleton for small EV proteins by our parent cells and listed in the ExoCarta database. Associations with all three pathways of the complement system were only found in the data set of more abundant NP small EV proteins via CLU, a secreted chaperone protein and inhibitor of the complement membrane attack complex (MAC) and therefore cell lysis (S6 Fig, S11 Table).

Mass spectrometry analysis of the FBS used here for cell culture suggested a list of 523 proteins (S12 Table). After removing these serum related proteins from our data sets, functional enrichment analysis was repeated for the 119 remaining more abundant NP small EV proteins (S7 Fig). This serum protein depleted dataset still identified complement and coagulation cascades (bta04610, KEGG M16894, WP M39649) and axon guidance (bta04360, KEGG M5539), but now also affiliated with longevity regulating pathway (bta04211), efferocytosis (bta04148), cellular senescence (bta04218) and more. The longevity connection was established through the RAS GTPases NRAS, KRAS, RHEB, and ADIPOQ, a plasma protein with anti-inflammatory and antioxidant effects [132] involved in AMP-activated protein kinase (AMPK) signaling (bta04152) for which we previously detected low level expression in NP cells cultured under comparable conditions (S3 Table) [105]. Efferocytosis enables tissue homeostasis following distinct phases of finding, binding, internalization and breakdown of apoptotic cells [133] and was identified through C1QA, C1QC; the solute carrier SLC16A1 involved in transport of monocarboxylates like pyruvate and lactate and driven by the proton motive force [134]; integrin subunit (ITGB5); the disintegrin and metalloproteinase domain-containing protein (ADAM10) and the small GTPase RAC1. Association with cellular senescence was also established through small GTPases. Transcripts were previously detected for all but C1Q, though some transcripts encoding Complement C1q Like proteins were expressed in NP cells (S3 Table) [105]. ToppFun analysis further identified, integrin pathway (PID M18), ECM organization (Reactome M610, MM14572) and hemostasis (M8925) amongst the top pathways (S12 Table). Interestingly, EPH-ephrin mediated repulsion of cells (Reactome, M27311), EPH forward pathway (PID M62) and EPH-ephrin signaling (Reactome, M27201) remained significant, essentially through the EPH receptor-RAS signaling axis and ECM components (S12 Table).

Similar associations were observed for all 300 NP small EV proteins after removing the serum proteins from the dataset. Additional pathways of interest like signaling by NOTCH4 (Reactome M954), regulation of RUNX3 expression (MM15536), somitogenesis (Reactome, M48031), and stabilization of P53 (Reactome M27670) were essentially based on an affiliation with the proteasome 20 subunits (S7 Fig, S12 Table).

## Discussion

Extracellular vesicle research is an evolving field, even more when it comes to cells of the IVD. Current technologies do not allow for proteomic analysis of IVD small EV cargo *in vivo, in situ.* Scaled-up cell culture remains inevitable at this time to study the NP small EV proteome. It is generally acknowledged that culture conditions can affect a cells’ secretome, which could cause data inconsistency. Discovery of novel or unique biomarkers associated with small EVs and/or parent cell lineages might have been obscured by the *in vitro* approach. To limit artifacts, the parent cells used here were exposed to identical media components and culture conditions, and experiments were conducted with biological and technical replicates, mitigating experimental bias as much as possible. We have investigated small EV proteins from autologous NP, AF and FAT parent cell sources as well as fetal NP tissue derived cells. Our data was compared to existing small EV data in a proteome centered approach backed up by previously acquired transcriptome data.

Amongst spine related health concerns IVDD is one of the major causes of lower back pain, a degenerative disease currently lacking a cure [6]. Regenerative stem cell therapies have been considered for IVDD; however, the harsh NP microenvironment poses a challenge. Small EVs, now regarded a tool of cell communication as part of many cells’ secretome, showed favorable therapeutic properties in various applications including increased cell proliferation, ECM synthesis, migration of repair-involved cells to an injury site, and promoted the differentiation of MSCs into NP cells while also showing reduced apoptosis, inflammation, ECM degradation, and cell senescence in NP cells [86,93,135]. A recent study highlighted the therapeutic potential of implementing a TIE2-positive (+) cell-enhancing protocol, emphasizing the importance of optimizing culture conditions. The EV product isolated from TIE2^+^ NP cells demonstrated promising regenerative outcomes, yet its cargo was not specifically characterized [97]. The focus of our analysis was to specifically investigate the NP small EV proteome for content and anticipated roles of NP small EV proteins in comparison to those of autologous AF and adipose cells, fetal NP cells and MSC. Our interpretation relays on multiple functional enrichment and pathway tools (DAVID, ToppFun, KEGG, STRING) with KEGG being more focused on disease related databases, ToppFun most up to date in its annotations and STRING considering known and predicted protein/protein interactions.

**Fig 10:**
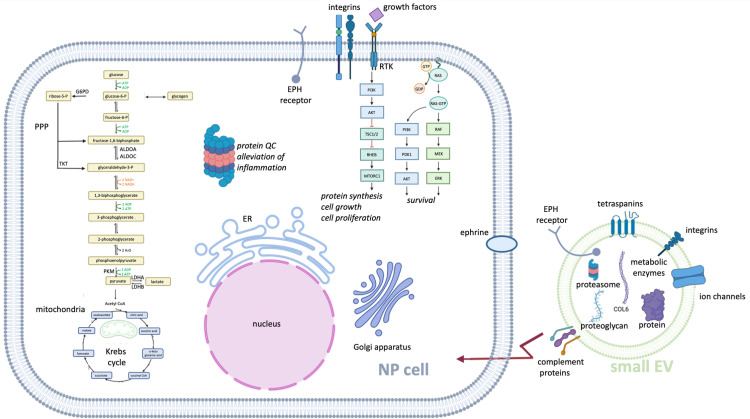
Multifaceted roles of the small EV proteome in NP cell and tissue homeostasis. NP small EV protein cargo and membrane constituents are involved in key metabolic pathways, including glycolysis, gluconeogenesis, Krebs cycle and PPP. They are crucial for energy production and to maintain the cellular redox balance. NP small EVs with the aid of the 20S proteasome, ensure protein quality control and reduced inflammation in a recipient cell. NP small EVs modulate signaling through EPH receptors, impacting cellular communication and tissue organization. Additionally, NP small EV interact with the complement system, influencing the classical, alternative, and lectin pathways involved in immune responses and inflammation. The PI3K/AKT/RAS signaling pathway is axis is impacted by the NP small EV proteome, which could promote ECM synthesis, cell growth and proliferation. Together, these processes underscore the essential role of the NP small EV proteome in sustaining NP cell function and NP niche homeostasis. ECM: Extracellular matrix; EPH: Ephrin receptor, ER: endoplasmic reticulum, ERK: extracellular signal-regulated kinase, MEK: mitogen-activated protein kinase, NP: nucleus pulposus, PDK1: phosphoinositide-dependent protein kinase 1, PPP: pentose phosphate pathway, PI3K/AKT: phosphoinositide 3-kinase/protein kinase B, RAF: rapidly accelerated fibrosarcoma, RHEB: RAS homolog enriched in brain, mTORC1: mammalian target of rapamycin complex 1, small EV: small extracellular vesicles, TSC1/2: tuberous sclerosis proteins 1 and 2. This illustration was created on Biorender. (www.biorender.com/).

Bovine coccygeal IVDs are an accepted model for young, healthy human IVDs enabling the study of cell homeostasis under non-diseased conditions [105]. While bovine small EVs might show species and/or tissue specific variations that could affect their makeup and functionality the extensive overlap with existing small EV data is supportive of this approach. The majority of small EV proteins identified here were contained in the ExoCarta and Vesiclepedia databases. Despite much of the existing small EV data being based on body fluids such as urine and serum, or cancer cell lines, we found significant overlap. While we have not discovered any novel NP biomarkers based on their small EV proteome, we believe that the NP small EV proteome cargo makes important contributions to NP cell and tissue homeostasis as outlined below (Fig 10).

### A potential for small EVs to engage with cell metabolism

Disc homeostasis depends on proper metabolism in the NP, which is linked to all cellular activities, and connected to various cellular dysfunctions, including degenerative changes such as IVDD [136]. An important energy source for NP cells is glucose, which can be used to generate ATP via glycolysis in anoxic conditions [137]. Degenerative changes in the CEPs or the AF could impact nutrient supply, resulting in cell stress and further progression of IVDD [136]. Cause-effect relationships between metabolic pathways and NP function remain under investigation [136,138]. Proteome profiling suggested a role of NP small EVs in glycolysis, gluconeogenesis, PPP and glutathione metabolism. Glutathione was previously considered as a potential treatment option for IVDD as it protects the IVD from oxidative stress [136,139]. Glycolytic enzymes were identified by small EV proteomic studies [140]. While these enzymes were not overrepresented among NP small EV proteins, we found several in our profiling data sets from fetal and adult NP parent cells, including LDHA, which has high affinity for pyruvate and preferentially converts pyruvate to lactate as an important step to restore the redox balance [141]. GAPDH, widely regarded as a housekeeping gene, fulfills diverse functions such as influencing cell fate by regulating cell death [142]. The contribution of functional glycolytic enzymes to a recipient cells’ energy and redox balance was suggested as one of the beneficial therapeutic outcomes of MSC small EV [140]. In addition to glycolytic enzymes TKT and G6PD were identified as part of the PPP. Their presence in NP small EVs suggests a form of metabolic coupling between parent and recipient cells possibly promoting various biosynthesis processes [136,143,144] and potentially equally beneficial than that of MSC small EV.

### Housekeeping via proteosome delivery

NP cells are resilient *in vitro*. The proteasome pathway was linked to small EV proteins of bovine parent cells, existing sEV data, generally associated with NP small EVs, and also more abundant in NP small EVs over parent cells. Proteasomes play crucial roles in cellular proteostasis maintaining functional protein quality and removing protein waste [145,146]. Proteasome subunits were detected in EVs present in the growth media of mammalian cells and in body fluids [147–162]. Studies suggested a parent cell specific encapsulation of proteasome subunits *in vitro* [148,162]. The exact mechanism by which proteasomes are targeted to EVs and their specific function within the EV context remains unclear, however, EVs facilitate cargo delivery to recipient cells through several mechanisms, including endocytosis, fusion to the plasma membrane, or phagocytosis [148,163,164]. Small EV proteasomes could maintain recipient cell proteostasis upon introduction into target cells. Alternatively, small EVs may also facilitate the export of proteasomes into the ECM to act on extracellular targets. For this, the content of the EVs should be emptied into the extracellular space. Studies have shown that lipid-degrading enzymes such as secretory phospholipase A2 and sphingomyelinase can dismantle EVs, liberating proteasomes into the extracellular environment [165]. Also inflammatory mediators could facilitate the release of lipid-degrading enzymes in response to cytokine activation, such as IL1, Interferon γ, and TNFα [166]. Potential therapeutic benefits of MSC small EVs with functional 20S proteasomes were demonstrated in a mouse model of myocardial ischemia, reducing the extent of damaged heart tissue and lowering levels of misfolded proteins [167]. Proteasomes in NP small EVs might therefore be instrumental for NP homeostasis and to alleviate inflammation.

### Immunomodulation in an immune privileged environment

The healthy NP is considered immune privileged due to the physical blood-NP barrier established by the dense lamellar structure of the AF and the PG-rich CEPs, resulting in an absence of blood and lymphatic vessels in the healthy adult NP [124]. Nerves and vessels however are present in its periphery. Immune suppressive molecular factors like the cell surface death receptor Fas ligand (FASLG) were described for the NP and NP cells *in vivo* [168,169]. We could not detect FAS or FASLG in NP cells or NP small EVs generated *in vitro* by proteome analysis, however *FAS* and low level *FASLG* transcripts were previously detected in cultured NP cells, alongside transcripts for the migration inhibitory factor (*MIF*) [105].

Small EVs were previously associated with coagulation, immune modulation and the complement system [170,171]. Small EV proteins from all sources investigated here associated with pathways of the (innate) immune system and neutrophil degranulation amongst others. Neutrophils are known as the most abundant type of immune cell in the blood. A connection between IVDD and the infiltration of immune cells including different neutrophil subpopulations was described previously [172,173]. Neutrophils generate small EVs [174] and small EVs likely diffuse into NP tissue from the periphery [175]. However, neutrophils have a short lifespan *in vitro* and their research generally depends on freshly isolated cells from blood [176]. The cells cultured here, though of low passage number, were rinsed profoundly prior to exosome collection and unlikely contained neutrophils or their small EVs. The complement system represents a group of distinct plasma proteins as part of the innate immune response, essentially complementing antibodies with opsonization by acting through the classic, alternative or lectin pathway [177]. Complement proteins and related pathways were predominantly associated with more abundant NP small EV proteins and present in existing data sets. While NP cells are not a classic source for complement proteins, dysregulation of complement proteins was previously linked to inflammatory diseases such as IVDD, CEP lesions, osteo- and rheumatoid arthritis and a potential connection to neovascularization and innervation [177]. We found some complement proteins in NP small EVs and previously detected transcripts for several in NP cells, many at very low levels though [105]*.* The association of NP small EV proteins with the (innate) immune or the complement system could therefore be attributable to a serum corona, however, this alone does not provide sufficient explanation for a persistent association with above mentioned pathways after eliminating FBS associated proteins from the proteome data set. Other pathway associations such as focal adhesion and regulation of the cytoskeleton could also be an *in vitro* artefact, as NP cells are known to be very motile in 2D culture [178].

### Receptor shuffling for niche homeostasis

Conserved during the evolution of domains and species, yet initially viewed as cellular debris, increasing evidence suggests that (small) EVs play a crucial role in the horizontal transfer of molecules between cells and as mediators of cell-cell communication, modulating mammalian cell signaling pathways such as PI3K/AKT, MAPK, AMPK, and cAMP which regulate a variety of cellular functions such as proliferation, survival, metabolism, and cell death [179–182].

Vesicle mediated transport and membrane trafficking were among the top pathways identified for bovine small EV proteins and existing databases. Small EVs are likely essential for promoting intercellular communication in the microenvironment of the IVD, facilitating a flow of bioactive molecules among disc cells to preserve tissue homeostasis. Small EVs could be instrumental in maintaining an avascular and aneural environment in the healthy NP. Recent work demonstrated the theoretical possibility for small EVs to travel through the dense ECM meshwork *in vivo* [175] and the described bidirectional exchange of membrane components by multisized vesicles during NP cell and MSC coculture [183] supports small EVs as communication form. More recently, small EVs isolated from NC cell conditioned medium showed transforming properties and small EVs isolated from MSC had positive effects on NP cells [107,184,185]. With a size limited capacity of small EVs, membrane surface proteins might be put in place by the parent cell prior to vesicle release [186]. Secretory vesicles such as small EVs are an important source of cell receptors [187]. Small EVs can interact with their target cells through cell surface receptors and signaling cascades, fusion with the target cell or via endocytosis [188]. Several of the here identified NP small EV proteins associated with EPH-RTK signaling [189,190]. EPH-ephrin domain compositions and complex forward (receptor) and reverse (ligand) signaling events are reviewed in great detail elsewhere [191–193]. This signaling pathway is known for its role during embryonic development including the partitioning of the dorsal mesoderm into NC and paraxial mesoderm [194–196], and its’ implication with tissue homeostasis and repair in the adult, maintaining skeletal, hematopoietic and neuronal integrity [192,197]. EPH-ephrin interaction typically requires cell-cell contact as receptor and ligand are membrane bound, allowing for uni- or bidirectional signaling [192]. Signaling robustness and strength is correlated with the extend of cluster formation [192,198]. *EPH receptor* and *ephrin ligand* transcripts were detected in NP cells *in vitro* [105,190], however only EPH receptors were identified in the NP small EV proteome. Endocytosis of EPH-ephrin complexes as a mean to regulate signaling was described [199]. Though EPH-ephrin signaling in the adult lacks behind its understanding during embryogenesis, it could be relevant for IVD homeostasis that this pathway is reestablished to control stem cell niches; cell homeostasis; tissue boundary formation; cell proliferation, differentiation and migration; metabolism and the cytoskeleton with an antiproliferative effect on the neural stem cell niche, inhibiting neurogenesis beyond just impacting axon guidance as reviewed in [192,197]. Furthermore EPH-ephrin, NOTCH and VEGF signaling play important roles in angiogenesis and arteriovenous patterning through the control of cell fate decisions [200]. Here we found an association between proteins of small EVs from NP parent cells and EPH-receptors. We therefore propose the following potential mechanisms based on qualitative and quantitative small EV proteomics data generated here as well as previous RNA transcript analysis in comparable 2D culture conditions [105] (S8 Fig): A) “Down-tuning” of signals in NP parent cells: Studies have suggested a function for EVs in removing undesired compounds, as such contributing to cellular homeostasis by protecting cells [201,202]. Packaging EPH receptors into small EV membranes could remove them from NP parent cells, preventing non-ligand induced forward signaling amidst plentiful growth factors in culture medium. This could prevent excessive proliferation [190,191]. B) “Amplifying” signals in target cells: On the contrary, shuffling EPH receptors to NP target cells could intensify a downstream forward signaling response in target cells. C) “Avoiding consequences” small EVs could circumvent an otherwise required close cell contact and enable a unique form of long-range communication still based on membrane contact by inducing reverse signaling in target cells without forward signaling consequence in the parent cell [198]. Future *in vivo* validation might favor or exclude one of the mechanisms.

In addition to EPH receptors, we identified many components of the RAS signaling cascades. It was suggested that RAS downstream signaling is important for small EV biogenesis, release, maintenance and signaling. RAS engagement in RTK signaling can lead to cell fate decisions between survival and death, essentially through the activation of the ERK/MAPK or PI3K/AKT pathways [193,203,204]. Other RTKs were identified as well, such as VEGFR2 which can activate downstream PI3K/AKT and mammalian target of rapamycin (MTOR) signaling, impacting angiogenesis via cell proliferation and survival [205]. The RTK epidermal growth factor receptor (ERBB1) downstream pathways can initiate the mitogen-activated protein kinase (MAPK) cascade or PI3K/AKT signaling [206]. The PI3K/AKT pathway, can influence cell proliferation, apoptosis, autophagy, and differentiation under physiological and pathological conditions by interacting with a number of downstream target proteins, including MTOR and Forkhead box O1 (FOXO1) [63,207]. Also triggered by RAS signaling events, the MAPK/extracellular signal-regulated kinase (ERK) pathway is an essential intracellular signal transduction pathway that is critical in maintaining IVD homeostasis [208–212].

### Maintaining ECM homeostasis

Harnessing “stemness” is an intriguing approach taken by the field of regenerative medicine to treat degenerative diseases or tissue loss. This can include the transplantation of transdifferentiated somatic cells, induced pluripotent stem cells or embryonic stem cells, all though posing a risk of tumorigenesis. Additionally, some cell types are deemed uneconomical on an individualized basis, are not fully understood in their differentiation potential, or face ethical concerns [213–216]. Autologous or allogeneic MSC gained popularity since their less-tumorigenic multipotent potential might be directed into the appropriate cell type via endogenous cues from the recipient tissue. However, transplanted stem cells often face delivery and survival challenges, especially in tissues with a naturally harsh microniche environment such as the IVD. MSC likely signal to resident stem cell populations via small EVs to kick-start refurbishment [185,217–219]. Given the adaption of NP cells to their niche, healthy NP parent cells seem a logic source for small EV production. Since no direct association of NP small EV proteins with stemness was identified, maintenance of cell and tissue homeostasis in the NP could be their main contribution.

During IVDD progression, not only is the expression of pro-inflammatory markers and ECM degrading enzymes noted, but also the exhaustion of progenitor cells. At the same time ECM synthesis reflected by structural collagens, Acan and small PGs declines as complex events of cellular senescence and regulated cell death rise [9,10,12,220–226]. ECM components like fibronectin connect with cell adhesion receptors and integrins to facilitate cell-cell and cell-matrix interactions, which have an impact on cell differentiation, proliferation, and function [227–232]. Integrin receptors too possess unique bidirectional signaling properties, coordinating extracellular events such as binding with laminin, or stiffness of the ECM with intracellular changes such as activation of the PI3K/AKT signaling pathway [233–247]. EV associated fibronectin plays a role in maintaining pluripotency and stemness of embryonic stem cells, while fibronectin fragments increase with IVDD progression [233,248,249]. These fragments may contribute to IVDD by inducing ECM degradation while suppressing PG synthesis [168,248,250–253]. Moreover, fibronectin is a significant heparan sulfate ligand mediating cell-small EV interactions and heparan sulfate proteoglycan 2 (HSPG2), a more abundant NP small EV protein, facilitates small EV uptake by cells [235].

Through interactions with fibronectin, laminin and other ECM molecules, collagen VI (COLVI) influences cell-ECM interactions, thereby modulating cellular behavior and tissue function [254–256]. COLVI which was more abundant in NP small EVs is a multifaceted protein associated with ECM organization, nervous system development, focal adhesion and PI3K/AKT-MTOR signaling, maintaining tissue and cell homeostasis [257]. It provides tensile strength and structural integrity to the ECM, and is involved in regulating apoptosis, reducing oxidative stress, and maintaining cell stemness [258]. By modulating signaling cascades involved in programmed cell death, COLVI maintains tissue integrity and function [257,259]. Its antioxidative properties scavenge reactive oxygen species (ROS), thereby protecting cells from oxidative damage [260,261]. In stem cell niches, COLVI maintains stemness by providing a supportive microenvironment. This maintains stem cell populations and supports tissue regeneration processes [257,259]. By modulating signaling pathways involved in cell cycle regulation and ECM production, COLVI promotes tissue homeostasis [259,262].

### Current limitations and outlook

We identified a considerable overlap in small EV proteins and function between our and existing data, supporting our approach. While proteome profiling of IVD derived small EVs by state-of-the-art mass spectrometry enabled the suggestion of proteome related molecular mechanisms behind cell and tissue homeostasis in this unique organ, small EV nucleic acid cargo was not assessed here. It remains to be elucidated which bioactive molecule class takes the center role in small EV mediated cell-cell communication. Further work and technology development will hopefully also provide insight into stoichiometric relationships between number of molecules per small EV surface or volume and minimum numbers of small EVs triggering phenotypic effects.

## Conclusions

Small EV research in the IVD recently gained momentum due to advantages over cell based regenerative therapies. This work focused on the proteomic profiling of small EVs, especially bovine NP small EVs. Under standard culture conditions, we found no clear indication that the here identified NP small EV proteins mobilized progenitor cells other than through the RAS signaling cascade; neither is there reason to believe they initiated senescence. Instead, the NP small EV proteome appeared to mediate NP niche and ECM homeostasis including cell fate decisions. We suggest that small EVs from NP parent cells contribute to NP cell metabolism and NP niche homeostasis beyond MSC by maintaining the unique avascular, aneural and immune privileged environment through anti-angiogenic and axon growth inhibitory processes. We believe that in the challenging niche environment, NP small EVs provide resources for an effective redox, energy and pH balance through the delivery of key metabolic enzymes. The association with EPH-RTK signaling further suggests that NP small EVs could regulate cell surface membrane receptor densities and therefore impact on the transduction of extracellular signals to the NP cell. Small EVs from NP parent cells might also deliver proteasome subunits, which could be of great importance in regulating crucial signaling cascades such as RUNX, NOTCH4, P53, PTEN, Hedgehog and others.

## Supporting information

S1 Fig Example of RNA *in situ* hybridization for some ECM markers. IVD tissue (top panels) and derived cell lines (bottom panels) were investigated for transcripts. Mallory’s tetrachrome stain was used in IVD tissue for histological reference. Bovine digoxygenin labeled RNA probes were generated through reverse transcription from polymerase chain reaction generated templates, employing the T7 promoter for the antisense probe. Collagen I (*Col1a1;* F:GGGGCAAGACAGTGATCGAA/ R: TTGGCTTTTCGGGGGTTTCA (229 bp)), collagen II (*Col2a1;* F:TCACAGAAGACCTCCCGTCT/ R:TCACAGAAGACCTCCCGTCT (561 bp)) and collagen VI (*Col6a1*; F: ACATCACCAAACGCTTTGCCA/ R: GGACAGAGAACCAGGTGCCA (821 bp)) are shown as example. Scale bar reflects 25μm. Red arrows indicate negative and green arrows indicate positive cells for gene expression. AF: annulus fibrosus, F: forward primer, IVD: intervertebral disc, NP: nucleus pulposus, R: reverse primer.
(TIF)

S2 Fig Original uncropped Western blot membranes. Focus areas for CD63 and TSG101 are indicated by a blue rectangle. 2k: DUC fraction containing debris and larger EVs; 10k: DUC fraction containing larger EVs; 130k pre: DUC small EV fraction before PBS wash; 130K post: DUC small EV fraction after PBS wash; CD63: Cell surface protein of the tetraspanin family; Hi-FBS: small EV fraction of heat inactivated fetal bovine serum used for cell culture prior to exosome harvest serving as small EV positive control. HRP: horseradish peroxidase; NP: nucleus pulposus; p5: passage 5 parent cell; M: New England Biolabs (NEB) marker; TSG101: Tumor susceptibility gene 101.
(TIF)

S3 Fig Functional enrichment analysis of autologous cells. A) Biological processes (BP); (B) molecular functions (MF) and (C) cellular components (CC) associated with 102 shared small EVs proteins from NP, AF and FAT parent cell lines; D) Biological processes (BP); (E) molecular functions (MF) and (F) cellular components (CC) associated with 156 small EV proteins from NP parent cells. AF: Annulus fibrosus; FAT: Adipose tissue; NP: Nucleus pulposus.
(TIF)

S4 Fig Functional enrichment analysis of adult and fetal NP and UCMSC cells. A) Biological processes (BP); (B) molecular functions (MF) and (C) cellular components (CC) associated with 206 shared small EV proteins from adult NP, fetal NP, and UCMSC parent cells. NP: Nucleus pulposus; UCMSCs: Umbilical cord mesenchymal stem cells.
(TIF)

S5 Fig Quantitative functional enrichment analysis of 141 proteins more abundant in NP small EVs as compared to their parent cells. A) Biological Processes (BP), B) Molecular Function (MF), C) Cellular Component (CC). NP: Nucleus pulposus.
(TIF)

S6 Fig Venn diagrams identifying shared ToppFun pathways for the top 200 pathways associated with the different small EV protein sources. AF: Annulus fibrosus; FAT: Adipose tissue; NP: Nucleus pulposus; or: overrepresented; UCMSC: Umbilical cord mesenchymal stem cell.
(TIF)

S7 Fig Comparison of fetal bovine serum proteins and small EV proteins by various NP parent cell sources. A) Comparison of small EV proteins from different NP parent cell sources with proteins in fetal bovine serum used for culturing. B) KEGG pathway analysis in DAVID for remaining NP small EV proteins from all sources combined and C) from more abundant NP small EV protein safter serum protein exclusion. D) ToppFun pathway analysis for remaining NP small EV proteins from all sources combined and E) from more abundant NP small EV proteins after serum protein exclusion.
(TIF)

S8 Fig Simplified schematic of complex EPH-ephrin signaling events. EPH: Ephrin receptor; EFN: ephrin ligand; R: receptor; L: ligand.
(TIF)

S1 Table Protein Concentration of small EV Fractions. NP: nucleus pulposus; TT32, TT33, TT39: cell lines; p: passage number.
(XLSX)

S2 Table Identified small EV proteins. AF: annulus fibrosus; FAT: adipose tissue; NP: nucleus pulposus.
(XLSX)

S3 Table Averaged TPM values for RNA sequencing data on NP, AF and FAT cells based on two (FAT) or three (AF,NP) replicates per cell line. The data generated under comparable conditions and depostited in NCBI GEO (https://www.ncbi.nlm.nih.gov/geo/), accession number GSE216377. TMP: Transcripts per million.
(XLSX)

S4 Table ToppFun functional enrichment analysis for pathways associated with bovine small EV proteins contained in ExoCarta and/or Vesiclepedia datasets showing a selection of the top 200 pathways. (XLSX)

S5 Table Functional enrichment analysis (DAVID) comparing small EV proteins of autologous bovine NP, AF, and FAT parent cells. AF: Annulus fibrosus; bta: Bos taurus; cAMP: Cyclic adenosine monophosphate; DAVID: Database for annotation, visualization, and integrated discovery; ECM: Extracellular matrix; FAT: Subcutaneous adipose tissue; GO: Gene ontology; KEGG: Kyoto encyclopedia of genes and genomes; FAT: Subcutaneous adipose tissue; MAPK: Mitogen-activated protein kinase; NP: Nucleus pulposus.
(XLSX)

S6 Table ToppFun analysis of shared pathways between small EV proteins from autologous bovine NP, AF, and FAT parent cells. AF: Annulus fibrosus; FAT: Subcutaneous adipose tissue; GO: gene ontology; bta: Bos taurus; cAMP: Cyclic adenosine monophosphate; ECM: Extracellular matrix; KEGG: Kyoto Encyclopedia of Genes and Genomes; FAT: Subcutaneous adipose tissue; MAPK: Mitogen-activated protein kinase; NP: Nucleus pulposus. *denotes 20 pathways detected for all investigated bovine parent cells in this study; ^#^denotes 9 pathways detected for all investigated small EV proteins in this study.
(XLSX)

S7 Table Functional enrichment analysis (DAVID) comparing small EV proteins of adult and fetal NP cells with UCMSC cells of shared exosome proteins of NP, fetal NP, and UCMSC cells. bta: *Bos taurus*; DAVID: Database for annotation, visualization, and integrated discovery; FAT: Adipose tissue; KEGG: Kyoto encyclopedia of genes and genomes; NP: Nucleus pulposus; UCMSC: Umbilical cord mesenchymal stem cells.
(XLSX)

S8 Table ToppFun analysis of shared pathways between small EV proteins from bovine adult and fetal NP, and UCMSC parent cells. bta: Bos taurus; ECM: Extracellular matrix; KEGG: Kyoto Encyclopedia of Genes and Genomes; NP: Nucleus pulposus. *denotes 20 pathways detected for all investigated bovine parent cells in this study; ^#^denotes 9 pathways detected for all investigated small EV proteins in this study.
(XLSX)

S9 Table Functional enrichment analysis (DAVID) after quantitative analysis of NP small EV proteins comparing NP small EV and parent cells. bta: *Bos taurus*; DAVID: Database for annotation, visualization, and integrated discovery; GO: Gene ontology; KEGG: Kyoto encyclopedia of genes and genomes; NP: Nucleus pulposus.
(XLSX)

S10 Table ToppFun pathway analysis of more or less abundant NP small EV proteins. (XLSX)

S11 Table Comparison of top 200 results of ToppFun pathways for each small EV source identifies nine pathways shared by all sources. AF: Annulus fibrosus; NP: Nucleus pulposus; UCMSC: Umbilical cord mesenchymal stem cells.
(XLSX)

S12 Table ToppFun pathway analysis for FBS proteins and all NP small EV proteins after FBS protein removal. FBS: Fetal bovine serum; NP: Nucleus pulposus.
(XLSX)

## Abbreviations

AF: annulus fibrosus
BLAST: basic local alignment search tool
BP: biological processes
CC: cellular components
CEP: cartilaginous endplates
CLC: chondrocyte like cells
DAVID: database for annotation, visualization, and integrated discovery
DLS: dynamic light scattering
DMEM: Dulbecco’s modified Eagles medium
DUC: differential ultracentrifugation
ECM: extracellular matrix
EPH: Ephrin receptor
ESCRT: endosomal sorting complex
EV: extracellular vesicle
FAT: adipose tissue
FBS: fetal bovine serum
FC: fold change
FDR: false discovery rate
GO: gene ontology
IVD: intervertebral disc
IVDD: intervertebral disc degeneration
KEGG: Kyoto Encyclopedia of genes and genomes
LC-MS/MS: liquid chromatography-tandem mass spectrometry
MCL: Markov clustering algorithm
MF: molecular function
MSC: mesenchymal stem cell
NC: Notochord
NP: nucleus pulposus
*p*: p-value
PG: proteoglycan
PID: Pathway interaction database
PPP: pentose phosphate pathway
PTM: posttranslational modification
RTK: receptor tyrosine kinase
SA: senescence associated
SEM: scanning electron microscopy
small EV: small extracellular versicle
STRING: search tool for the retrieval of interacting genes/proteins
UCMSC: umbilical cord mesenchymal stem cells
WB: Western blot.
