# Supplementary figures and images for: Proteomic profiling of small extracellular vesicles from bovine nucleus pulposus cells
Source: PLoS One. 2025 May 29;20(5):e0324179. doi: 10.1371/journal.pone.0324179 (PMC12121814; doi:10.1371/journal.pone.0324179)

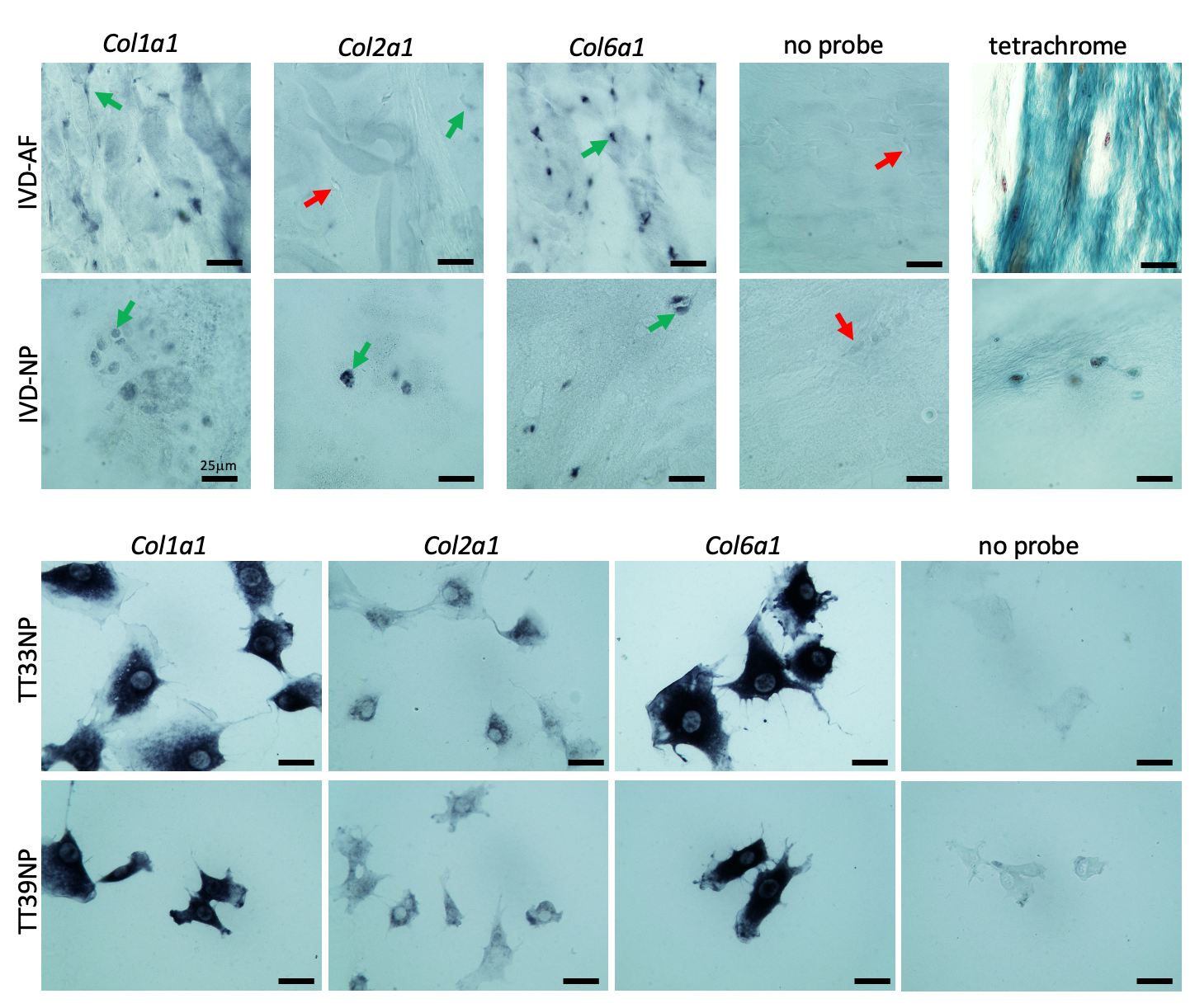

Supplement: S1 Fig — IVD tissue (top panels) and derived cell lines (bottom panels) were investigated for transcripts. Mallory’s tetrachrome stain was used in IVD tissue for histological reference. Bovine digoxygenin labeled RNA probes were generated through reverse transcription from polymerase chain reaction generated templates, employing the T7 promoter for the antisense probe. Collagen I (Col1a1; F:GGGGCAAGACAGTGATCGAA/ R: TTGGCTTTTCGGGGGTTTCA (229 bp)), collagen II (Col2a1; F:TCACAGAAGACCTCCCGTCT/ R:TCACAGAAGACCTCCCGTCT (561 bp)) and collagen VI (Col6a1; F: ACATCACCAAACGCTTTGCCA/ R: GGACAGAGAACCAGGTGCCA (821 bp)) are shown as example. Scale bar reflects 25μm. Red arrows indicate negative and green arrows indicate positive cells for gene expression. AF: annulus fibrosus, F: forward primer, IVD: intervertebral disc, NP: nucleus pulposus, R: reverse primer. (TIF) [file pone.0324179.s001.tif]

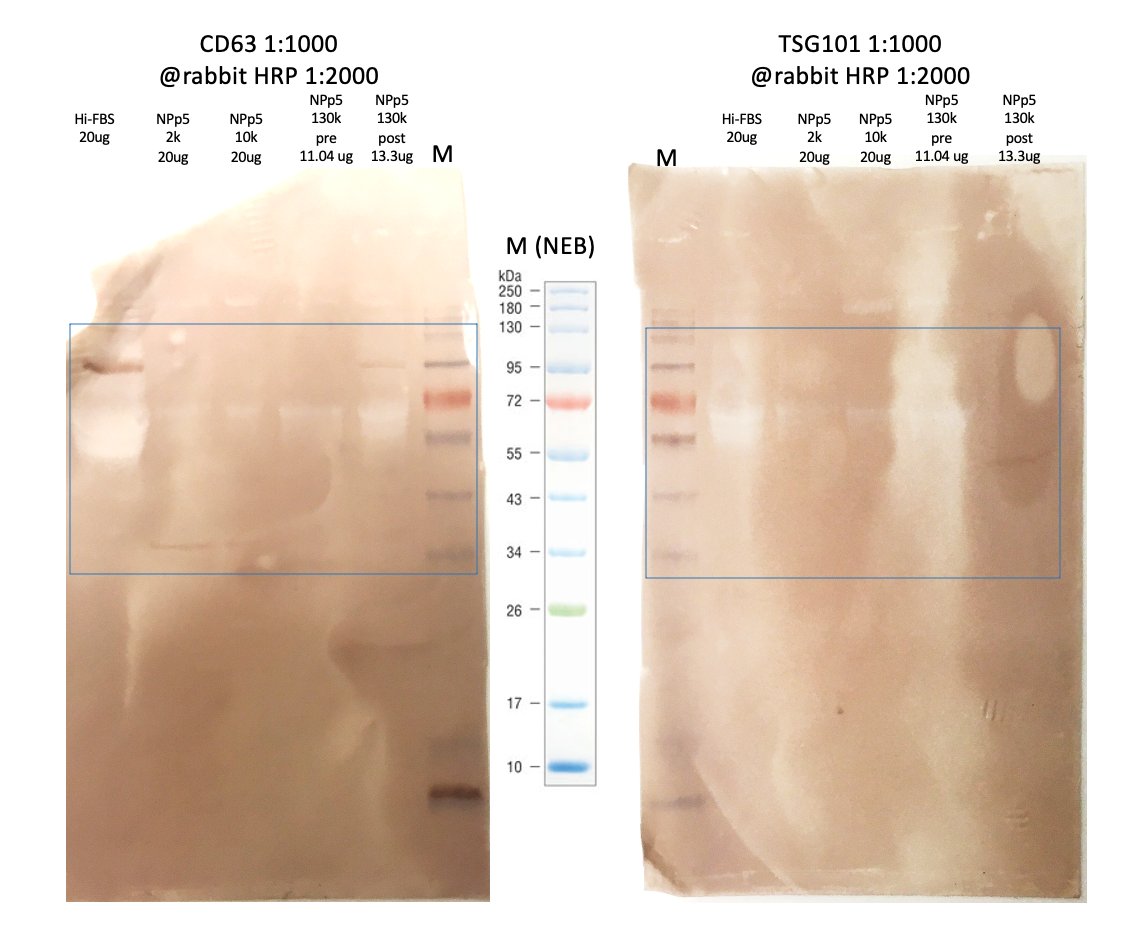

Supplement: S2 Fig — Focus areas for CD63 and TSG101 are indicated by a blue rectangle. 2k: DUC fraction containing debris and larger EVs; 10k: DUC fraction containing larger EVs; 130k pre: DUC small EV fraction before PBS wash; 130K post: DUC small EV fraction after PBS wash; CD63: Cell surface protein of the tetraspanin family; Hi-FBS: small EV fraction of heat inactivated fetal bovine serum used for cell culture prior to exosome harvest serving as small EV positive control. HRP: horseradish peroxidase; NP: nucleus pulposus; p5: passage 5 parent cell; M: New England Biolabs (NEB) marker; TSG101: Tumor susceptibility gene 101. (TIF) [file pone.0324179.s002.tif]

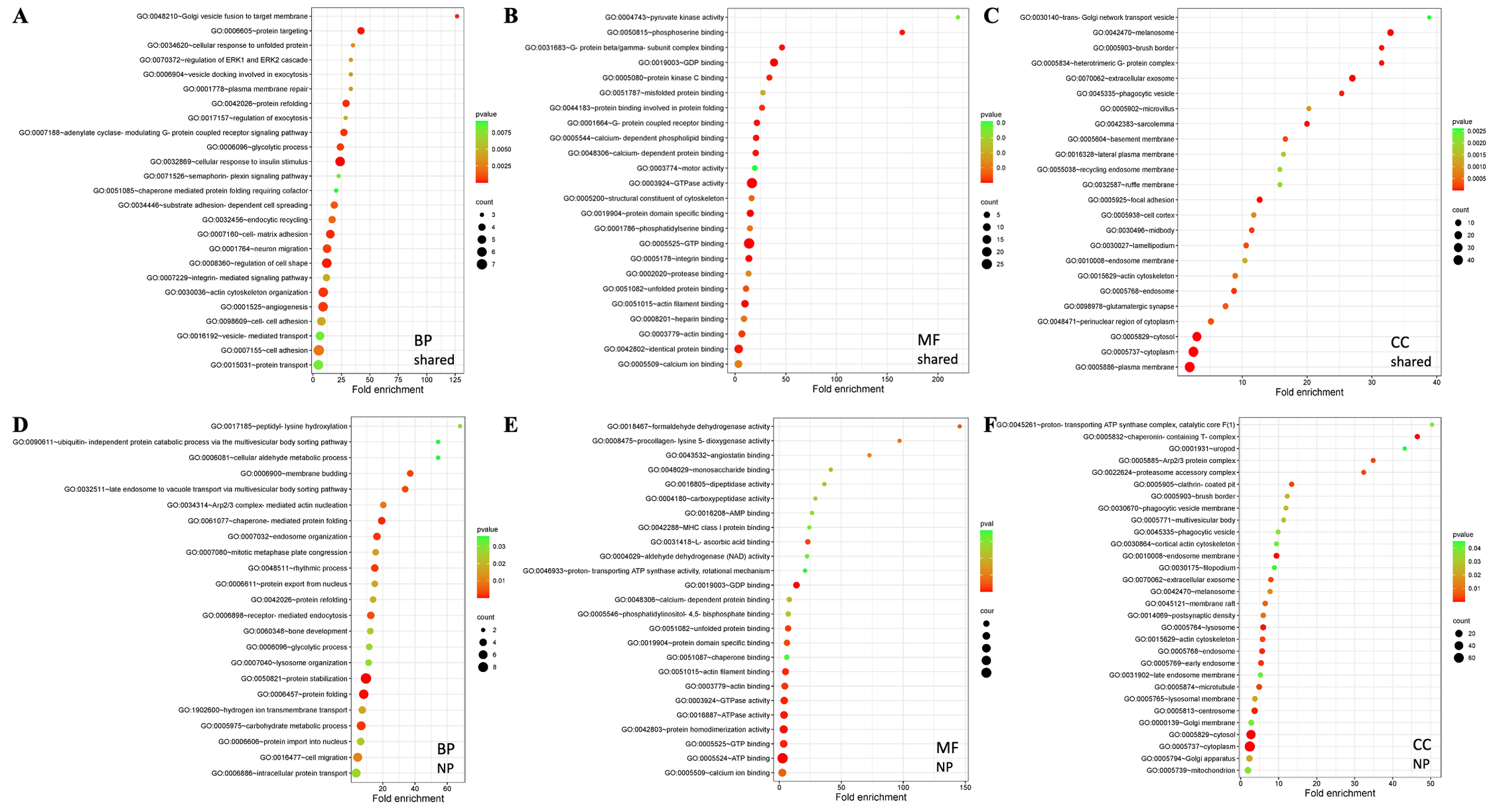

Supplement: S3 Fig — A) Biological processes (BP); (B) molecular functions (MF) and (C) cellular components (CC) associated with 102 shared small EVs proteins from NP, AF and FAT parent cell lines; D) Biological processes (BP); (E) molecular functions (MF) and (F) cellular components (CC) associated with 156 small EV proteins from NP parent cells. AF: Annulus fibrosus; FAT: Adipose tissue; NP: Nucleus pulposus. (TIF) [file pone.0324179.s003.tif]

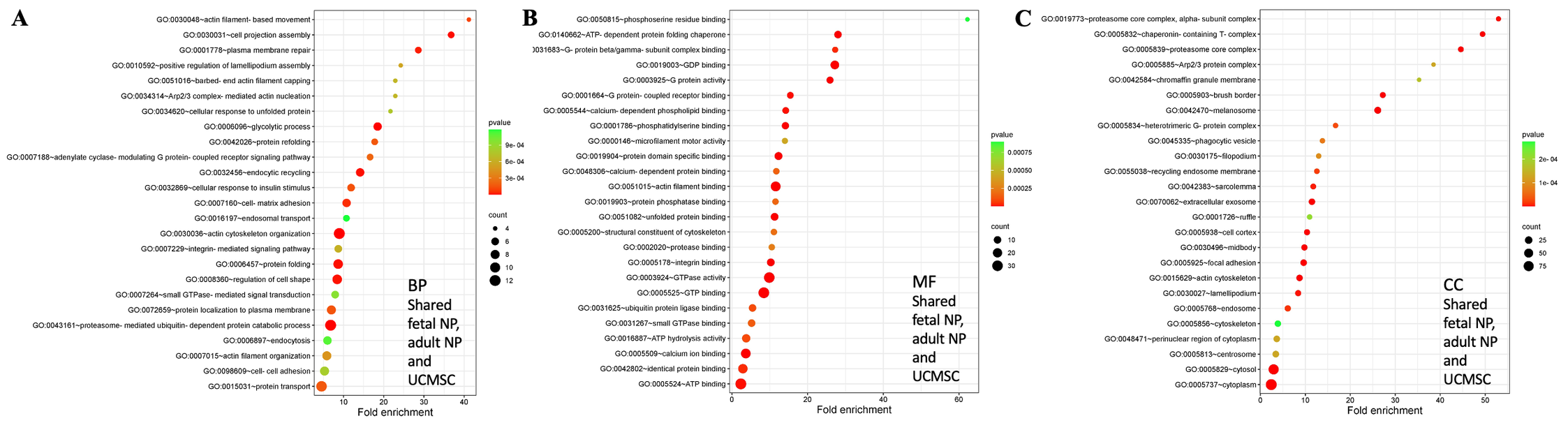

Supplement: S4 Fig — A) Biological processes (BP); (B) molecular functions (MF) and (C) cellular components (CC) associated with 206 shared small EV proteins from adult NP, fetal NP, and UCMSC parent cells. NP: Nucleus pulposus; UCMSCs: Umbilical cord mesenchymal stem cells. (TIF) [file pone.0324179.s004.tif]

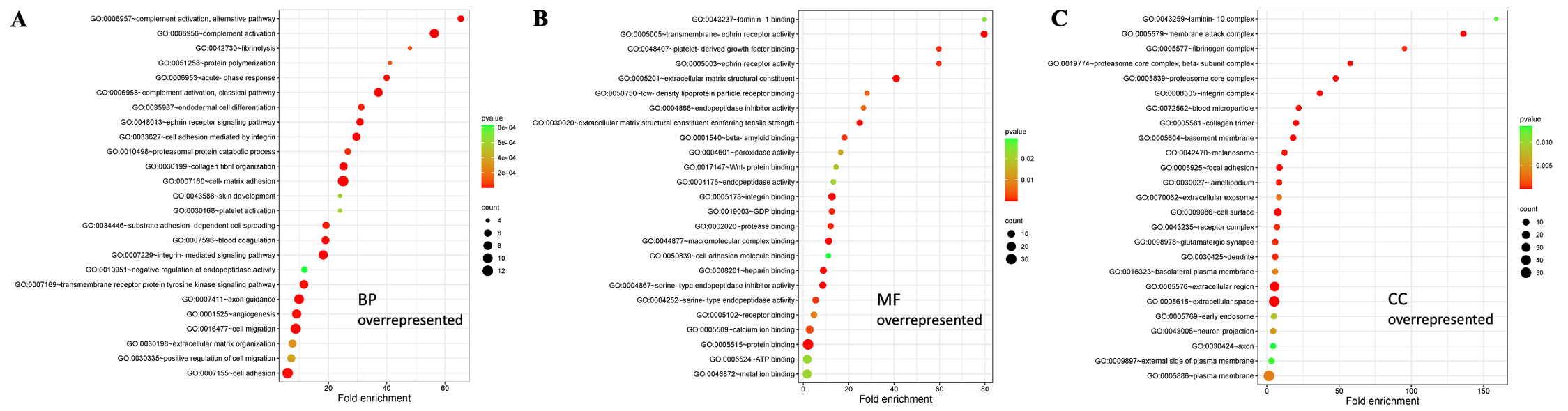

Supplement: S5 Fig — A) Biological Processes (BP), B) Molecular Function (MF), C) Cellular Component (CC). NP: Nucleus pulposus. (TIF) [file pone.0324179.s005.tif]

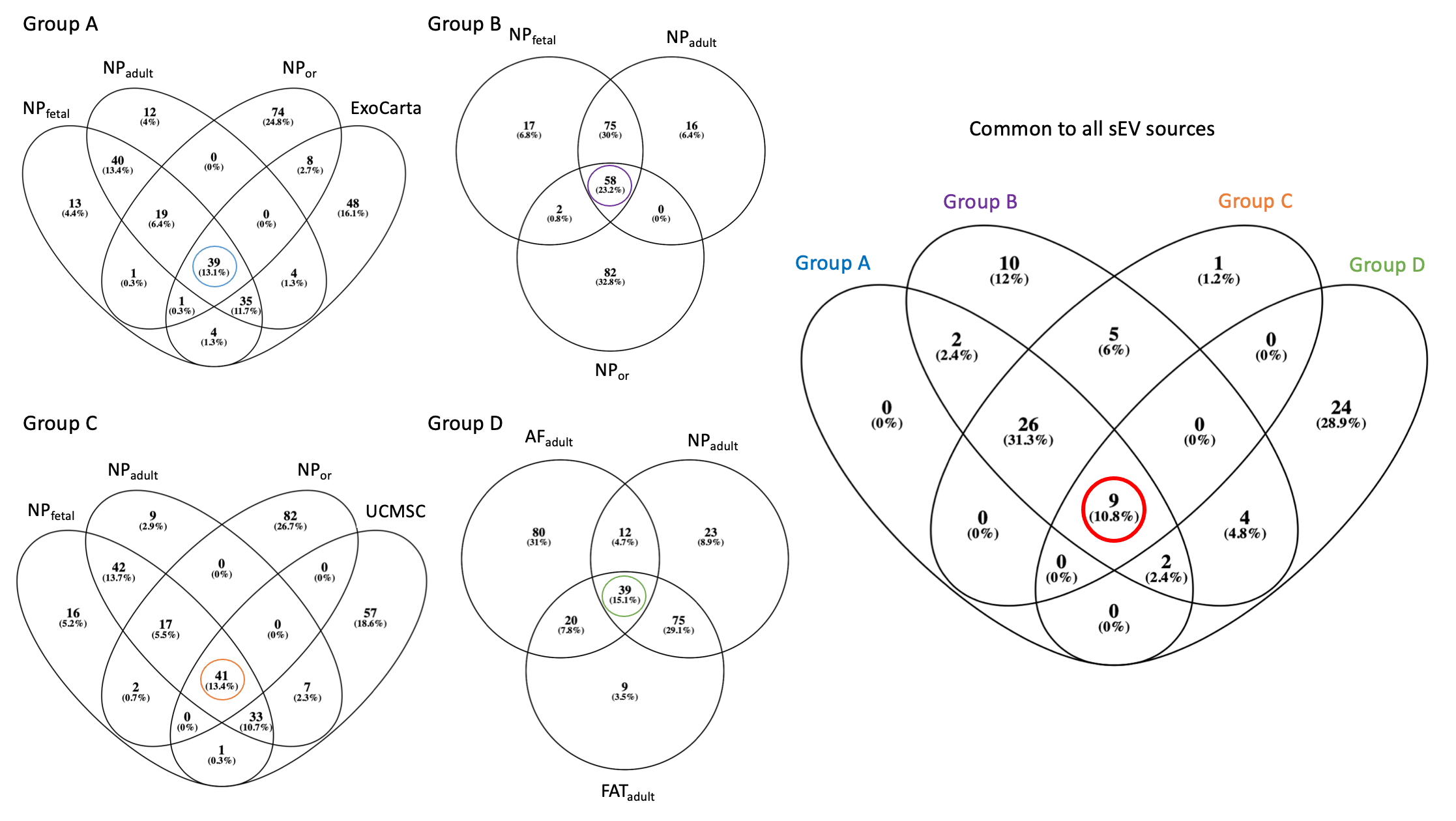

Supplement: S6 Fig — AF: Annulus fibrosus; FAT: Adipose tissue; NP: Nucleus pulposus; or: overrepresented; UCMSC: Umbilical cord mesenchymal stem cell. (TIF) [file pone.0324179.s006.tif]

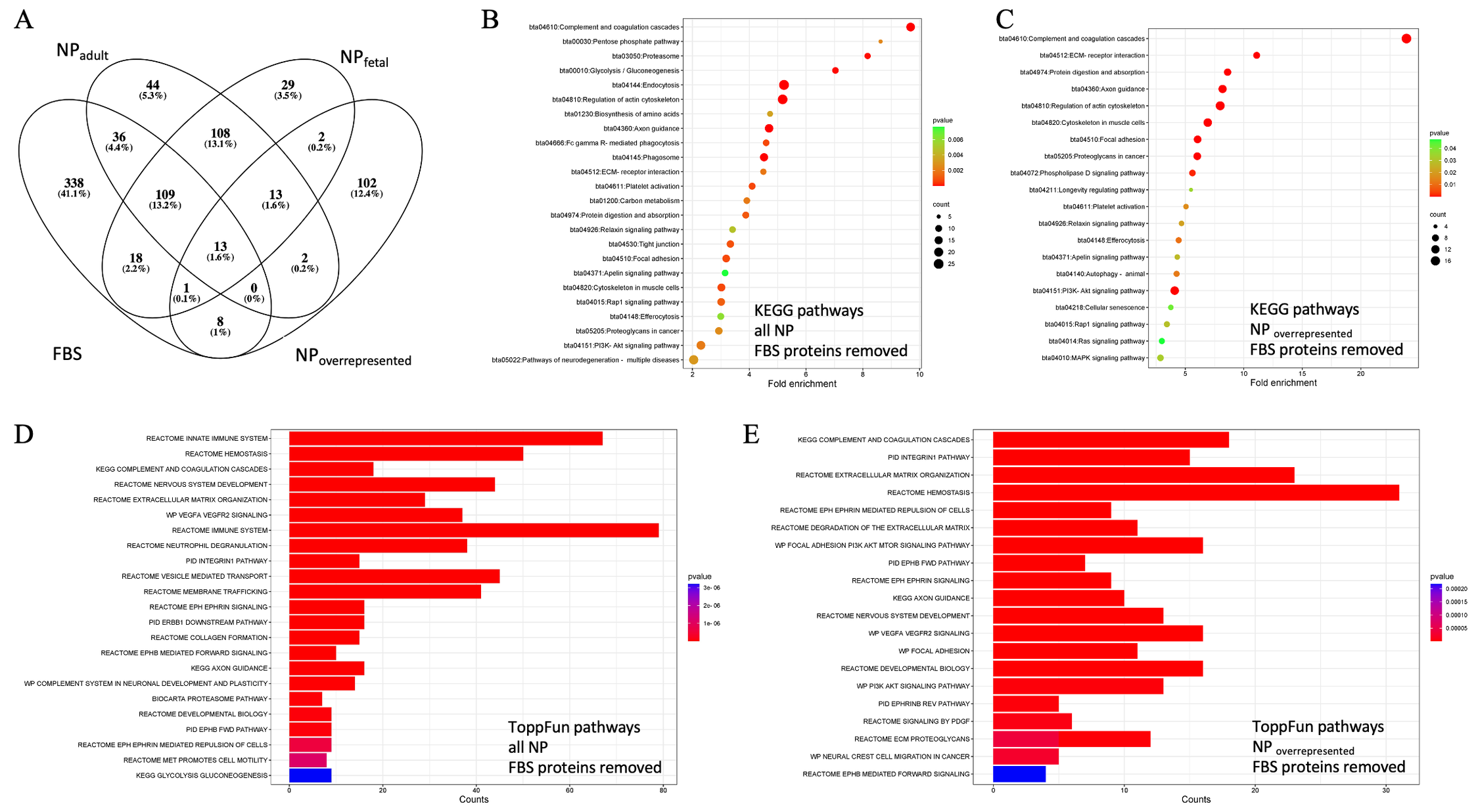

Supplement: S7 Fig — A) Comparison of small EV proteins from different NP parent cell sources with proteins in fetal bovine serum used for culturing. B) KEGG pathway analysis in DAVID for remaining NP small EV proteins from all sources combined and C) from more abundant NP small EV protein safter serum protein exclusion. D) ToppFun pathway analysis for remaining NP small EV proteins from all sources combined and E) from more abundant NP small EV proteins after serum protein exclusion. (TIF) [file pone.0324179.s007.tif]

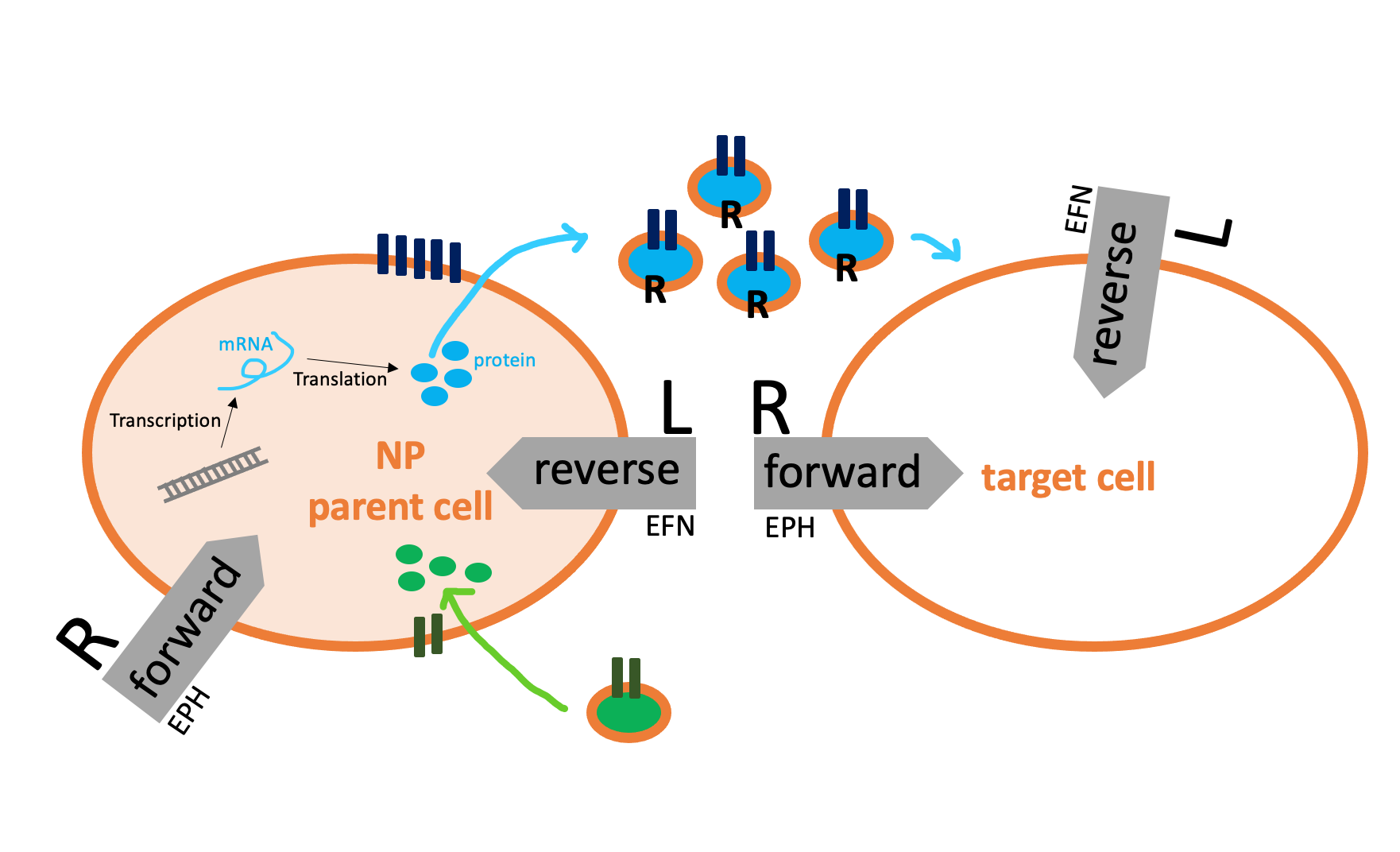

Supplement: S8 Fig — EPH: Ephrin receptor; EFN: ephrin ligand; R: receptor; L: ligand. (TIF) [file pone.0324179.s008.tif]
